# Multiscale characterization and distribution of the thyroid hormone transporter MCT8 in the adult and aged human brain

**DOI:** 10.1186/s40478-026-02435-3

**Published:** 2026-10-01

**Authors:** Jonas Rotter, Toni Kirmann, Jana Brendler, Larissa Anthofer, Nina Maria Wilpert, Stefan Hallermann, Heike Biebermann, Ingo Bechmann

**Affiliations:** 1 Institute of Anatomy, Leipzig University, Leipzig, Germany; 2 Carl-Ludwig-Institute of Physiology, Leipzig University, Leipzig, Germany; 3 Charité Universitätsmedizin Berlin, Corporate Member of Freie Universität Berlin and Humboldt-Universität zu Berlin, Institute for Experimental Paediatric Endocrinology, Berlin, Germany; 4 Department of Paediatric Neurology, Charité-Universitätsmedizin Berlin, Corporate Member of Freie Universität Berlin, Humboldt-Universität zu Berlin, and Berlin Institute of Health (BIH), Berlin, Germany; 5 NeuroCure Cluster of Excellence, Charité-Universitätsmedizin Berlin, Corporate Member of Freie Universität Berlin, Humboldt-Universität zu Berlin, and Berlin Institute of Health (BIH), Berlin, Germany; 6 Berlin Institute of Health at Charité-Universitätsmedizin Berlin, BIH Biomedical Innovation Academy, BIH Charité Junior Clinician Scientist Program, Berlin, Germany; 7 LeiCeM – Leipzig Center of Metabolism, Leipzig University, Leipzig, Germany

**Keywords:** Thyroid hormone transport, MCT8, Alzheimer's disease, Blood–brain barrier, Human brain, STED microscopy

## Abstract

The local availability of thyroid hormones (TH) depends on TH transporters such as monocarboxylate transporter 8 (MCT8, encoded by *SLC16A2*). Mutations of *SLC16A2* cause Allan-Herndon-Dudley syndrome (AHDS), a disorder characterized by severe neurodevelopmental and motor deficits. While MCT8’s importance in foetal brain development is well-established, its precise topographical, cellular, and subcellular distribution in the adult human brain has remained largely uncharacterized, limiting our understanding of which structures depend on MCT8-mediated TH transport throughout life and whether neurodegenerative processes such as Alzheimer’s disease (AD) affect local transporter availability. In this study, we systematically mapped MCT8 expression across eight functionally distinct brain regions from 14 aged human body donors. Our multiscale approach combined immunostaining, automated whole-slide image analysis of over 13 million cells, multiplex RNAscope in situ hybridization, stimulated emission depletion (STED) microscopy, and single-nucleus RNA sequencing data analysis. We additionally applied standardized Braak staging to investigate whether AD progression correlates with alterations in MCT8 expression. We demonstrate robust MCT8 expression across brain barriers, glial cells, and in distinct neuronal populations. RNAscope confirmed a strong concordance between *SLC16A2* mRNA presence and MCT8 immunoreactivity. STED and confocal microscopy localized MCT8 to plasma membranes, perinuclear regions, and axonal/dendritic compartments, indicating roles in both trans- and intracellular TH transport. MCT8 expression was preserved across Braak stages, with no significant difference between donors with varying degrees of neurofibrillary pathology. Our findings establish a comprehensive cellular and regional framework for MCT8-mediated transport in the human brain, mapping its specific anatomical distribution across regions crucial for motor control and cognitive processing. Stable MCT8 expression across Braak stages suggests that cerebral TH dysregulation in AD likely reflects altered hormone metabolism rather than transporter loss.

**Supplementary Information:**

The online version contains supplementary material available at https://doi.org/10.1186/s40478-026-02435-3.

## Introduction

Thyroid hormones (TH) have a crucial impact on the development and maintenance of physiological functions in the human brain. To enter various target cells in the brain, the blood–brain barrier (BBB) and cellular plasma membranes need to be crossed [6], requiring TH transporters such as the monocarboxylate transporter 8 (MCT8), the organic anion transporting polypeptide 1C1 (OATP1C1) and multiple others [42, 93].

Among the different TH transporters, MCT8 is of particular importance due to its high specificity for TH [42]. During human development, MCT8 provides the principal route for TH delivery to the foetus, as it regulates TH transport across the placenta and ensures adequate TH supply to the developing brain parenchyma [17, 38, 54]. Within the foetal brain, this is reflected by widespread expression of MCT8 at the BBB, in leptomeningeal cells, the hippocampus, and the brainstem [52]. The importance of foetal MCT8-mediated TH transport is illustrated by MCT8 deficiency, also known as Allan-Herndon-Dudley Syndrome (AHDS) [42, 53, 73]. In AHDS, mutations in the MCT8 encoding gene *SLC16A2* cause severe global developmental delay and movement disorders in affected patients due to local cerebral TH deprivation [29, 34, 73]. The severe clinical manifestations of AHDS emphasize the critical need for proper TH availability in early neurodevelopment, consistent with the severe neurodevelopmental deficits observed in conditions that limit overall foetal TH availability, such as congenital hypothyroidism and maternal iodine deficiency [4, 19, 49, 95].

Throughout life, TH continues to affect neurogenesis [24, 32], myelination [50], and brain metabolism [46]. Dysfunction in its adequate supply leads to neuropsychiatric disorders, reduced adult neurogenesis, and may be associated with dementia, memory loss and impaired attention [18, 68]. Corresponding to these diverse effects, MCT8 expression in the adult human brain has been described at brain barriers, including blood vessels and choroid plexus cells [3, 67, 91]. Its expression has been extensively studied in the hypothalamus, where it is present in both glial and neuronal cells in the arcuate, supraoptic, infundibular and paraventricular nuclei [3, 58]. Moreover, MCT8 has been reported in tanycytes [3], a type of specialized nutrient-sensing ependymal cells which send long processes deep into hypothalamic nuclei [10, 11, 37]. Recently, work by Wang and colleagues described the expression of MCT8 in pericytes, pyramidal neurons, subtypes of GABAergic interneurons, astrocytes and (myelinated) neurofilaments in the adult human motor cortex, basal ganglia and thalamus, while noting that microglia express OATP1C1, but not MCT8 [88, 89]. Highlighting the functional relevance of MCT8 for adult human cognition, common polymorphisms in *SLC16A2* have been associated with inter-individual variation in cognitive domains such as attention, alertness, and planning in healthy adults [80]. However, the actual role of MCT8 for maintaining adult and aged brain TH homeostasis and its transcriptional regulation remains unknown [42]. Although essential for understanding MCT8 in normal physiology as well as in aging and disease, a comprehensive characterization across brain regions and cell types is still missing.

Given this link to adult cognition, MCT8 is of particular interest in the context of Alzheimer’s disease (AD). AD is a multifaceted neurodegenerative disorder and the most prevalent form of dementia that mainly affects the brain, causing a progressive decline in memory, language, visuospatial skills, and overall cognitive function [57]. Epidemiological studies have linked abnormal TH levels to AD, although individual studies failed to draw a conclusive answer in a definite direction: some report lower TSH and TH levels in AD patients [22, 83, 94], others higher levels [26], and others no association [25]. Meta-analytic evidence points toward hypothyroidism as the more consistent finding, with a significantly higher prevalence of hypothyroidism in AD patients than in controls [71]. Whether this reflects a risk factor or a consequence of AD remains to be resolved in longitudinal studies. Mechanistic studies point to candidate pathways, including T3-dependent regulation of AD-related genes and TH-dependent modulation of microglial immune signalling, though their relevance to human AD pathophysiology remains to be established [31, 51]. Data on how MCT8 expression affects AD progression or vice versa are sparse. In a streptozotocin-induced sporadic AD rat model, TH supplementation improved cognitive impairment and attenuated glial neuroinflammation, accompanied by increased MCT8 expression, suggesting that MCT8 may be conditionally upregulated in response to elevated T3 [74].

In this study, we addressed the knowledge gap of MCT8 expression across the human brain. By combining immunolabelling, RNAscope, automated image analysis, STED microscopy, and single-nucleus RNA sequencing (snRNA-seq) data analysis, we comprehensively characterized the regional, cellular, and subcellular distribution of MCT8. We further assessed whether MCT8 expression changes with neurofibrillary pathology by analysing donors across Braak stages.

MCT8-expressing cells were present in all analysed regions, with distinct regional patterns in neuronal and non-neuronal cell populations and the highest neuronal proportions in the motor cortex and medulla oblongata. Detailed analyses across cortical and subcortical brain regions further identified region-specific expression patterns. Together, these findings provide the first comprehensive anatomical map of MCT8 expression in the adult and aged human brain, as well as an anatomical framework that may help explain the motor and cognitive impairments observed in individuals with MCT8 deficiency.

## Materials & methods

### Human tissue

All tissue samples used in this study were obtained from body donations received by the Institute of Anatomy in Leipzig. Use of these samples was approved by the Ethics Committee of the University of Leipzig (129–21 ek), in accordance with the Saxonian Death and Funeral Act of 1994, third section, paragraph 18, item 8. All donors provided written informed consent. After post-mortem examination and approval for further use, fresh tissue was obtained from different organs, including brain, thyroid gland and liver. Irrespective of gender, a total of 14 individuals with an age range of 64 to 101 years (mean 88.0 ± 9.0; median 88.0) and a post-mortem interval (interval between death and tissue retrieval) of 13–36 h were examined (Table S1). We dissected brain samples representing complementary functional systems: motor function (primary motor cortex/precentral gyrus and cerebellum), sensory processing (occipital cortex), higher-order association and executive function (frontal and temporal cortex), learning and memory (hippocampus and entorhinal cortex), and homeostatic and autonomic regulation (hypothalamus and medulla oblongata). These regions also show varying susceptibility to neurodegenerative pathology, enabling assessment of MCT8 expression across both phylogenetically conserved and cortical structures.

Depending on the downstream application, tissue samples were processed either as fresh-frozen or paraffin-embedded tissue blocks. For standard immunolabelling, fresh tissue was placed into cryomolds (Tissue-Tek Cryomold), covered in OCT (Tissue-Tek O.C.T.), frozen on dry ice-chilled isopentane (ROTH), and wrapped in aluminium foil for storage in air-sealed containers at − 80 °C. Fresh-frozen blocks were cryosectioned at 12 µm thickness (Leica CM3050 S). The sections were then mounted on Superfrost Plus slides (Epredia), dried for 5 min at room temperature, and stored at − 80 °C until further use.

For RNAscope in situ hybridization and immunolabelling comparison, tissue was fixed with 4% phosphate-buffered formaldehyde (Roti-Histofix) for 24 h, washed with tap water and then further processed for paraffin embedding (dehydrated through a graded alcohol series, cleared in xylene, and embedded in paraffin). Paraffin blocks were sectioned on a microtome at 12 µm thickness (Microm HM 400), mounted on Superfrost Plus slides (Epredia), dried overnight at 37 °C, and stored at room temperature.

For optimal MCT8 staining performance, various methods were tested, including different antigen retrieval protocols (citrate buffer, pH 6.0; TRIS buffer, pH 9.0), fixation types (100% ethanol, 4% phosphate-buffered formaldehyde) and embedding techniques (sucrose-protected cryopreserved, paraffin, polyethyleneglycole, OCT-fresh-frozen). We found that the epitope accessibility of MCT8 was best preserved when using fresh-frozen or shortly formaldehyde-fixed (≤ 24 h) tissue, as previously described [91].

### Antibodies and immunolabelling

For fresh-frozen tissue immunolabelling, the sections were placed in a − 20 °C freezer for 30 min and subsequently for 15 min in a 4 °C refrigerator for a slow equilibration process. The sections were fixed with 100% ethanol for 20 s and washed in cuvettes containing 0.02 M phosphate-buffered saline (PBS) at pH 7.4. To prevent non-specific binding, normal donkey serum (NDS) at a concentration of 1:20 in PBS containing 0.3% Triton (PBS-T) was applied for 30 min, matching the host species of the secondary antibody. After a further washing step, the sections were incubated overnight at 4 °C with the primary antibodies (Table S2) in 0.03% PBS/Triton and 0.5% NDS.

The next day, several washing steps were followed by incubation with the secondary antibody (Table S3) for 60 min at room temperature, DAPI staining and treatment with TrueBlack (Biotium TrueBlack, Cat#23,007) for 15 s to block autofluorescence. Finally, the sections were coverslipped with Dako Glycergel mounting medium (Dako, Cat#C0563).

To compare staining performance and investigate potential topological or conformational differences in subcellular MCT8 labelling (e.g. due to suspected N-terminal capping/oligomerization [40]), we evaluated four MCT8 antibodies targeting distinct epitopes. These included three polyclonal antibodies—one N-terminal (Sigma-Aldrich Cat#HPA003353, RRID: AB_1079343), one C-terminal (Novus Cat#NBP2-57,308, RRID: AB_3342370) and a middle region antibody (Antibodies online Cat#ABIN1387834, RRID: AB_3750275) – as well as one monoclonal antibody directed against the N-terminus (Abcam Cat#ab302706, RRID: AB_3750276) (Table S2). The polyclonal N-terminal and C-terminal antibodies had been validated previously [91]. We reconfirmed their specificity alongside the two additional antibodies using wild-type and MCT8 knockout mouse tissue collected during our previous study [91] (Fig. S1). The comparative staining performance of the four antibodies in human tissue is reported in the Results (Fig. S2). Based on this comparison, the polyclonal N-terminal antibody was used for all regional and cellular MCT8 distribution analyses, including the automated whole-slide quantification. For the RNAscope and STED microscopy experiments, we used both the N-terminal and the middle region antibody, as the latter showed the most intense intracellular signal (Fig. S2c, d). The peak-to-peak quantification was based on the middle-region antibody alone. Additionally, double- and triple-stainings were performed using cell-type- or hallmark-specific antibodies to investigate various study-specific questions (Table S2). Omission controls, in which the primary antibody was omitted while all other steps were kept unchanged, were run in parallel for every primary antibody to check for unspecific staining. Antibodies that did not yield interpretable signal in fresh-frozen tissue are listed separately in Table S2. Each antibody combination used for cellular characterisation was applied to sections from at least four body donors and two brain regions and yielded comparable and reproducible results.

### RNAscope

RNAscope analyses were performed using the Multiplex Fluorescent Reagent Kit v2 (ACDbio, Cat#323,100) following the manufacturer’s standard protocol. Tissue samples were fixed in 4% buffered formaldehyde for 24 h and embedded in paraffin. Slides were baked at 60 °C for 1 h, deparaffinized, and subjected to antigen retrieval in retrieval buffer in a lightly boiling water bath for 15 min. Endogenous peroxidase activity was blocked by incubating slides in hydrogen peroxide. Protease Plus (ACDbio, Cat#322,381) treatment was applied, followed by PBS washes. Hybridization was carried out by incubating sections with 1:50 diluted probe buffer (Table S4) for 2 h at 40 °C in a HybEZ™ II manual hybridization oven (ACDbio, Cat#321,710). Amplification and washing steps were performed in alternating order three times, followed by application of the tyramide signal amplification (TSA) fluorophores (1:750, Table S3, Opal 570/690).

Subsequent IF staining was performed by blocking non-specific binding with normal goat serum (NGS, 1:20 in PBS-T) for 2 h at room temperature, and incubating sections overnight with primary antibodies diluted in PBS-T containing 1% NGS. After several PBS-T washes, secondary antibodies diluted in PBS-T with 1% NGS were applied for 2 h at room temperature. Nuclear counterstaining was performed using DAPI for 12 min, and autofluorescence was quenched with True Black for 15 s. Finally, sections were coverslipped with Dako Fluorescence Mounting Medium (Dako, Cat#S3023).

### Stimulated emission depletion (STED) microscopy

Super-resolution imaging was performed using an Expert Line Abberior Confocal and STED microscope equipped with an Olympus UPlanXApo 60× oil objective (numerical aperture of 1.42). The microscope system included a pulsed high-power STED laser module operating at a wavelength of 775 nm, with a laser power of 3 W and a repetition rate of 40 MHz and 3 pulsed fluorescence excitation laser modules for wavelengths of 485, 561, and 640 nm. MCT8 and Lamin B1 (labelled with Abberior StarOrange and StarRed, respectively; see Supplementary Table S3) were sequentially imaged in two-dimensional STED mode with a 20 nm pixel size. Nuclei were stained with NucSpot® Live 488 (Supplementary Table S5) and imaged in confocal mode. All channels were captured with a dwell time of 5 μs/pixel and a two-fold line accumulation, with system settings kept strictly constant across all experiments.

To quantify spatial relationships, fluorescence intensity line profiles across the nuclear lamina were extracted from the STED images using ImageJ (Fiji). For each nucleus (10 nuclei in total across two biological samples), multiple line profiles (three to five) were drawn across the MCT8 and Lamin B1 signals. Peak positions for each channel were determined by fitting a Gaussian curve to the intensity profiles, enabling the calculation of the peak-to-peak distance between MCT8 and Lamin B1. Multiple measurements per nucleus were averaged to obtain a single representative value per nucleus. Because this analysis was designed to visually map subcellular spatial relationships rather than perform statistical testing, the data are reported descriptively. We present the distribution of all individual measurements alongside the nucleus-level means.

### Braak staging

For each brain donor (Table S1), neuropathological staging of neurofibrillary degeneration was performed according to Braak and Braak [12, 13]. Hyperphosphorylated tau in neurofibrillary tangles (NFTs) was detected by immunostaining using the AT8 antibody (Table S2). Donors were categorized as Control (Braak stages 0-I, minimal or absent NFTs confined to the transentorhinal cortex), Braak Low (stages II, NFTs in the entorhinal cortex), Braak Medium (stages III-IV, NFTs in limbic areas, including the hippocampus), and Braak High (stages V-VI, NFTs in neocortical areas) (Table S1). For the primary comparison, Control and Braak Low donors were combined into a "Control" group and Braak Medium and High donors into an "AD" group. Amyloid-β deposition was not staged in this cohort. The "AD" group designation therefore reflects the extent of neurofibrillary degeneration and does not constitute a neuropathological diagnosis of AD according to current consensus criteria.

### Microscopy, semi-quantification and statistical analysis

Confocal images were either captured with an Olympus FV1000 laser scanning microscope and Fluoview software (Olympus; pixel size ranging from 0.10 µm to 0.62 µm/pixel) or an Olympus BX51 epifluorescence microscope connected to an Olympus XM10 digital camera and CellSens software (pixel size ranging from 0.16 µm to 0.64 µm/pixel).

For the quantification analyses, a Zeiss AxioScan slide scanner in combination with Zeiss ZEN Software 3.7 was used to create high resolution full slide scans at 20× magnification (pixel size: 0.346 µm/pixel). Scans were then imported to QuPath 0.4.3 and processed using a custom written script (see Availability of data and material). We used StarDist (version 0.4) for nuclei recognition and subsequently performed fluorescence signal thresholding by calculating the mean fluorescence intensity for each given cell and adding four standard deviation values to distinguish MCT8 signals from background. Depending on the colocalization of DAPI, NeuN, and MCT8 signals, a total of 13,664,502 cells were classified in a total of 78 slides. To map regional MCT8 hotspots, cell coordinates were converted into grey-scale density maps in QuPath using a 500-pixel Gaussian kernel downsampled to 20 µm resolution (see visual workflow in Fig. S3). In FIJI (version 1.54), we applied a standard “Fire” Lookup Table to the density maps and merged them with low-resolution images of the original scans to colocalize morphological hallmarks with MCT8 expression hotspots. The raw cell classification data was exported for further processing using a Python (version 3.9) script calculating the number of classified and detected cells in each slide. We normalized these numbers by calculating the percentage of MCT8-positive cells within their respective target cell populations (total, neuronal, or non-neuronal). Where more than one section of a region was available from the same donor, the corresponding values were averaged so that each donor contributed a single value per region. Statistical comparisons were therefore performed on donor-level values. Regional differences were assessed separately for each of the three target populations using the non-parametric Kruskal–Wallis test followed by Dunn's multiple comparisons test, with p-values adjusted for all 28 pairwise comparisons within each analysis. Differences in MCT8 expression between the consolidated Control and AD groups were assessed using Mann–Whitney U tests. Differences across the four Braak subgroups were analysed separately for each cell class using two-way ANOVA with Braak subgroup and brain region as factors. Statistical analyses and graph creation were performed in GraphPad Prism (version 11.0.0, GraphPad Software, Boston, MA, USA). Information on the distribution of the raw whole-slide scan images can be found in the Data availability statement.

### Gene expression analysis

To investigate *SLC16A2* expression across human brain regions and cell types, we analysed publicly available snRNA-seq data from the human whole-brain transcriptomic cell type atlas [75], accessed through the Allen Brain Cell (ABC) Atlas (dataset WHB-10Xv3, release manifest 20260415). The dataset comprises over 3 million single-nucleus transcriptomes sampled across around 100 anatomical dissections of the adult human brain from three neurotypical donors, with cell-type, neurotransmitter, and anatomical annotations provided in the atlas metadata [75]. Analyses were performed in Python using abc_atlas_access for data retrieval, together with Scanpy (v1.11.1), NumPy (v2.2.5), Matplotlib (version 3.9.4) and Seaborn (v0.13.2).

Expression was quantified from the raw (unnormalized) count matrices. A nucleus was classified as *SLC16A2*-positive if it carried more than one raw *SLC16A2* UMI (i.e. ≥2 counts, to exclude background signal). Proportions of positive nuclei were computed (i) for each anatomical region x cell type, (ii) across major neuronal and non-neuronal cell classes, and (iii) across neurotransmitter-defined neuronal classes from the atlas annotation (each class reflects the primary neurotransmitter, with co-release combinations assigned to the primary class in the following order: histaminergic > dopaminergic > cholinergic > serotonergic > glycinergic > GABAergic > glutamatergic). Cellular proportions of *SLC16A2*-positive nuclei were calculated across anatomical regions, cell types, and neurotransmitter classes, excluding groups with <20 nuclei. To confirm that positivity calls (i.e. classifying a nucleus as MCT8-positive) were not biased by sequencing depth or single-cell dropout, we performed robustness checks against normalized thresholds (${\mathrm{log}}_{2}\left[CP10k+1\right]>1$), depth-explicit Pearson residuals (*θ =100*), and fine-cluster pseudobulk aggregation (*CPM>1.0*).

## Results

In this study, we characterized the spatial distribution patterns of MCT8 expression in adult and aged human brain tissue using a multi-scale analytical approach spanning four organizational levels: (1) cellular microenvironmental context, (2) cell type-specific distribution, (3) cytoarchitectural organization, and (4) regional comparative analysis across brain areas. To achieve this, we performed systematic immunohistochemical analysis of 78 brain tissue samples obtained from 14 individuals (Table S1), representing eight anatomically and functionally distinct brain regions.

### MCT8 antibody staining performance

To assess antibody specificity, all four MCT8 antibodies were validated in MCT8-knockout tissue, in which none produced a specific signal (Fig. S1). In human brain tissue, the staining performance varied significantly by antibody. The C-terminal antibody produced only weak signals (Fig. S2a). In contrast, the polyclonal N-terminal gave the strongest and most consistent staining, showing both membranous and perinuclear signals (Fig. S2b). Antibodies targeting the middle region primarily labelled the perinuclear compartment but also showed immunoreactivity in ramified cells (Fig. S2c, d). The monoclonal N-terminal antibody showed robust signals with cellular staining profiles similar to its polyclonal counterpart (Fig. S2e). Specificity was further supported by *SLC16A2* mRNA to protein colocalization (Fig. 3c). We therefore selected the polyclonal N-terminal antibody as the primary antibody of this study. The assignment of antibodies to the individual experiments is given in the Methods.

### MCT8 expression in non-neuronal cells of the human brain

**Fig. 1:**
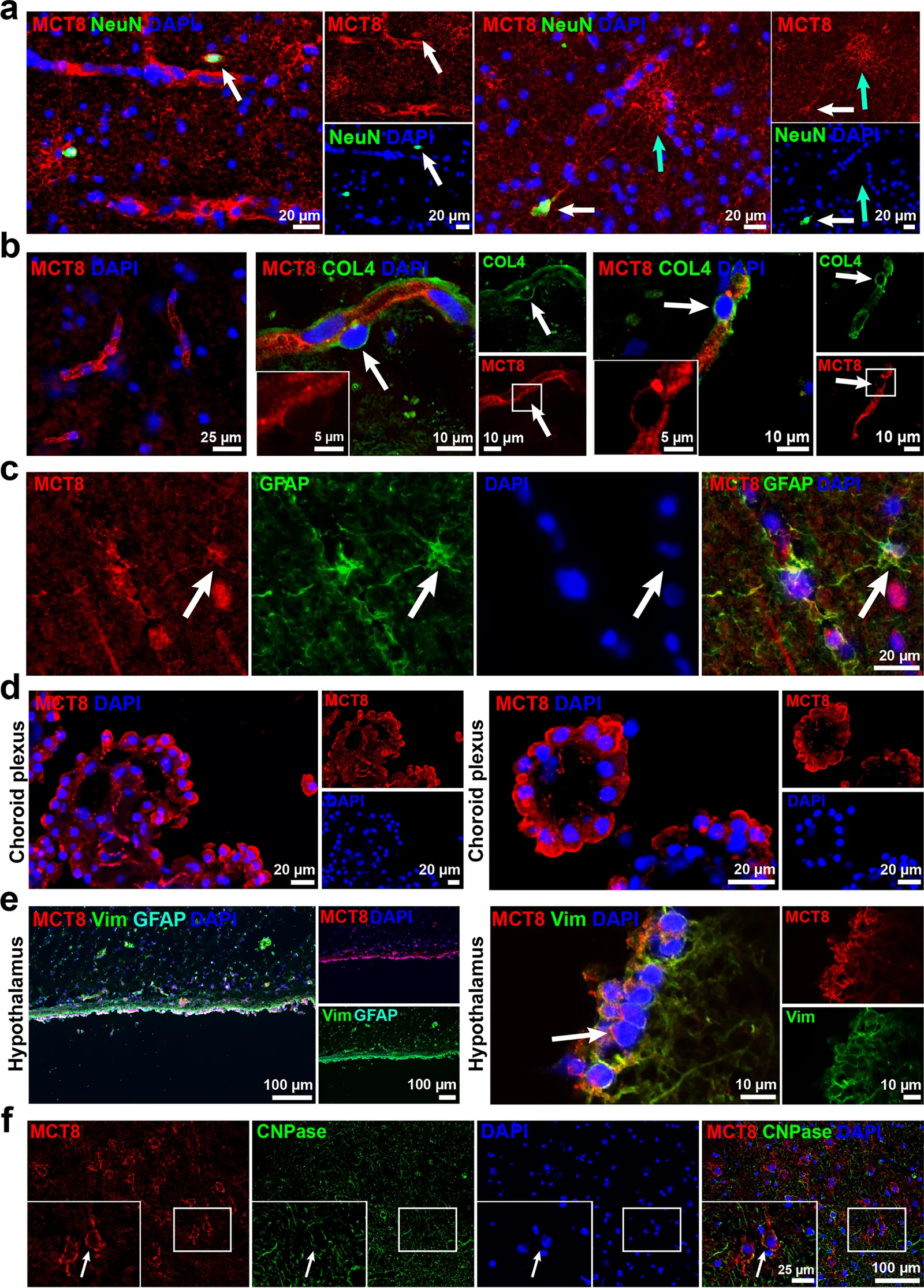
MCT8 is expressed at brain barriers and in other non-neuronal cells of the human brain. Panels a-e show the brain barrier-forming compartments (cerebral blood vessels, choroid plexus epithelium, tanycytes of the third ventricle), panel f shows oligodendrocytes. **a** MCT8 (red) expression in its cellular microenvironment. MCT8/NeuN (green), temporal cortex, two representative fields. MCT8 immunosignals outlined blood vessels and ramified parenchymal cells with their processes (cyan arrow, right panel), while NeuN-positive neuronal somata (white arrows) were scattered in the surrounding parenchyma. **b** Cerebral blood vessels. Left: MCT8/DAPI, hippocampal formation, showing MCT8-ir along the entire vascular profile. Middle and right: MCT8/COL4 (green) double stainings, hypothalamus, with enlarged views (insets). Arrows mark perivascular cells in a pericyte-like position, identified by local bulges of the COL4-immunoreactive basement membrane, without (middle) and with (right) a detectable MCT8 signal. **c** Astrocytes. MCT8/GFAP (green), hippocampal formation. GFAP-positive astrocytic processes adjacent to the vascular surface exhibited MCT8-ir (white arrows). **d** Choroid plexus of the lateral ventricle, MCT8/DAPI, at two magnifications. MCT8 signals were concentrated at the apical membrane of the choroid plexus epithelium facing the ventricular lumen, with weaker signals in the stromal core of the villi. **e** Tanycytes at the wall of the third ventricle, hypothalamus. Left: MCT8/Vimentin (green)/GFAP (cyan)/DAPI overview of the tanycyte layer lining the lateral ventricular wall. Anatomical location and marker combination are consistent with α-tanycytes. Right: High-magnification MCT8/Vimentin. MCT8 was present in the cell somata, at the apical surface and in the proximal basal processes (white arrow) and diminished as the processes extend into the hypothalamic parenchyma. **f** Oligodendroglial cells. MCT8/CNPase (green)/DAPI, hippocampal formation. Insets show enlarged views of the boxed regions. Colocalization was sparse. Most CNPase-positive somata lacked MCT8-ir. Occasional double-positive cells were seen (white arrows in the inset). Nuclei were labelled with DAPI (blue) in all images. Scale bars are indicated in each panel. *Abbreviations*: *COL4* Collagen IV, *GFAP* Glial fibrillary acidic protein, *Vim* Vimentin, *CNPase* 2',3'-cyclic nucleotide 3'-phosphodiesterase

Consistent with previous studies, we found abundant expression of MCT8 at brain barriers, with strong MCT8 immunoreactivity (MCT8-ir) in endothelial cells, perivascular cells, astrocytes, choroid plexus epithelial cells and tanycytes lining the third ventricle (Fig. 1). Beyond these barrier-forming compartments, MCT8-ir sparsely colocalized with oligodendrocytic structures.

To separate neuronal from non-neuronal MCT8 signals and to place them in their anatomical context, we first assessed the spatial relationship of MCT8-ir structures within the cellular microenvironment using MCT8/NeuN double stainings (Fig. 1a). MCT8-ir outlined blood vessels and was occasionally present in ramified cells of the adjacent parenchyma. Some of the processes of these cells approached the vascular surface. NeuN-positive neuronal somata were scattered between the MCT8-ir vessels at variable distance, sometimes directly adjacent to them. However, we did not observe MCT8-ir processes directly connecting neuronal somata with the vascular surface. Processes contacting blood vessels instead originated from NeuN-negative ramified cells (Fig. 1a, right image, upper white arrow).

To assess MCT8 expression in cerebral blood vessels, we performed double-stainings using MCT8 and collagen IV (COL4) antibodies. MCT8-ir outlined the vascular profile along its entire length (Fig. 1b, left). Along these vessels, the COL4-immunoreactive basement membrane formed local bulges enclosing individual cell bodies, outlining them towards both the endothelium and the surrounding parenchyma. This position within the vascular basement membrane is characteristic of pericytes [48, 55]. However, since none of the pericyte-selective markers we tested yielded interpretable staining in our fresh-frozen material (Table S2), we refer to these cells as perivascular cells in a pericyte-like position. Part of these perivascular cells displayed MCT8-ir surrounding the soma, whereas others showed no detectable MCT8 signals (Fig. 1b, enlarged details).

In addition to the rather homogenous, almost cylindrical signals that allowed for blood vessel identification, we found distinct cellular processes showing MCT8-ir contacting the outer surface of the blood vessels. GFAP-MCT8 double stainings identified these processes as astrocytic (Fig. 1c). Within the grey matter, only a limited number of GFAP-expressing astrocytes showed MCT8-ir, typically at vascular contacts. In contrast, MCT8-ir astrocytes were more frequently located within the subcortical and juxtacortical white matter, where they were situated amidst axonal structures.

In the choroid plexus, we observed strong MCT8 IF signals at the epithelial cell membrane facing the ventricle (apical surface) (Fig. 1d). Furthermore, we detected less intense signals at the inner side of the villus, which we morphologically identified as MCT8-positive stromal capillaries.

Along the wall of the third ventricle, Vimentin-positive cells with basal processes extending into the hypothalamic parenchyma showed MCT8-ir (Fig. 1e). Their Vimentin immunoreactivity and radial morphology identified these cells as tanycytes. They were located in the ventricular wall, and a substantial proportion of them co-expressed GFAP along Vimentin (Fig. 1e, left) – a combination of location and marker profile reported for α-tanycytes [39]. We therefore classified these cells cautiously as α-tanycytes. As the median eminence itself was not represented in the analysed sections, β-tanycytes were not sampled and no statement can be made regarding MCT8 expression in this subtype. At higher magnification, MCT8 signals were present in the tanycyte somata and at their apical surface facing the ventricle and continued into the basal processes (Fig. 1e, right) but diminished within a few hundred micrometres as the processes extended into the parenchyma.

To assess MCT8 expression in oligodendrocytes, we performed co-IF stainings for MCT8 and 2',3'-cyclic nucleotide 3'-phosphodiesterase (CNPase), a marker of differentiating and mature oligodendrocytes [79]. We observed only sparse colocalization of MCT8 signals with CNPase-positive cell bodies and proximal processes (Fig. 1f). Most CNPase-positive somata lacked detectable MCT8-ir and prominent MCT8-positive somata were typically CNPase-negative. We did not observe MCT8-ir in cells with microglial morphology. As the microglia-specific antibodies available to us did not perform adequately in the fresh-frozen tissue (Table S2), we addressed this cell type separately by RNAscope instead (Fig. 3). Both cell types are quantified in the single-nucleus transcriptomic dataset presented below (Fig. 7).

### MCT8 expression in neurons

MCT8-ir was consistently observed in cells with neuronal morphology. In addition, long, fibre-like structures in the juxtacortical and subcortical white matter exhibited MCT8-ir. To confirm their neuronal identity, we performed double stainings with several neuron-specific antibodies (neuronal nuclei (NeuN), microtubule-associated protein 2 (MAP2), neurofilament heavy chain (NF-H)) and subsequently employed markers of neurochemically defined neuronal populations, including glutamate decarboxylase 67 (GAD67), tyrosine hydroxylase (THy) and vesicular glutamate transporter 1 (VGLUT1). A systematic analysis of populations defined by neurotransmitters is provided by the snRNA-seq analysis presented below (Fig. 7).

**Fig. 2:**
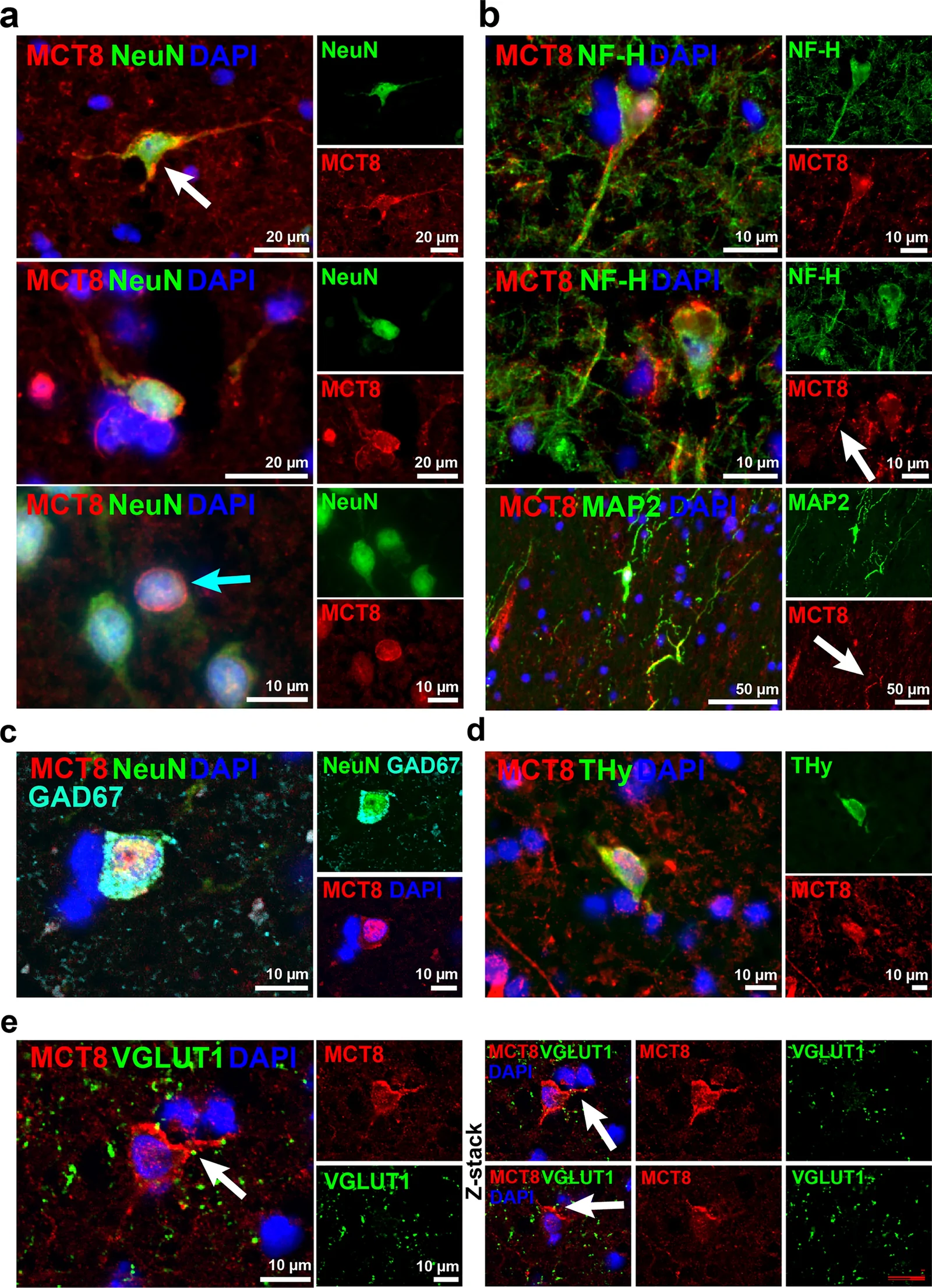
MCT8 immunoreactivity in neurons of the human brain. **a** MCT8 (red) and NeuN (green) double staining in the temporal cortex. MCT8 immunoreactivity (MCT8-ir) varied in intensity and distribution: some neurons exhibited a membrane-like staining pattern that surrounded the soma and extended into proximal processes (white arrow), while others showed a predominantly perinuclear pattern (cyan arrow). **b** MCT8 (red) double staining with the axonal marker NF-H (green; top and middle rows, temporal cortex) and the somatodendritic marker MAP2 (green; bottom row, hippocampal formation). MCT8-ir colocalized with NF-H-positive axons and MAP2-positive dendrites. Arrows indicate the punctate distribution of the MCT8 signal along the labelled processes. **c** MCT8 (red), NeuN (green) and GAD67 (cyan) triple staining in the motor cortex. Right: merged NeuN/GAD67 (top) and MCT8/DAPI (bottom) channels of the same field. A NeuN- and GAD67-positive neuron shows co-expression with MCT8. **d** MCT8 (red) and THy (green) double staining in the parahippocampal gyrus. A THy-immunoreactive neuron exhibits MCT8-ir. **e** MCT8 (red) and VGLUT1 (green) double staining in a z-stack of the same cell (z-step 1 µm) in the hippocampal formation. VGLUT1-positive presynaptic puncta are closely adjacent to an MCT8-ir neuron (white arrows). These images indicate the apposition of glutamatergic terminals to an MCT8-expressing neuron, rather than co-expression of MCT8 and VGLUT1 within the same cell. Nuclei were labelled with DAPI (blue) in all images. Scale bars are indicated in each panel. *Abbreviations*: *NF-H* Neurofilament heavy chain, *MAP2* Microtubule-associated protein 2, *GAD67* Glutamate decarboxylase 67, *THy* Tyrosine hydroxylase, *VGLUT1* Vesicular glutamate transporter 1

Double staining for MCT8 and NeuN confirmed neuronal MCT8 expression across all examined brain regions and revealed two major patterns of neuronal MCT8-ir (Fig. 2a). The first pattern was observed in large neurons with prominent membranous MCT8 signals encircling the cell soma and extending into both apical and basal dendrites. These neurons were predominantly found in the hippocampus, temporal cortex, and motor cortex, whereas fewer were observed in the frontal and occipital cortex. The second pattern was associated with smaller neurons exhibiting less extensive MCT8 immunosignals. Some of these smaller neurons appeared solitary, while others were located among larger pyramidal neurons (see also Figs. 5 and 6). Both types of neurons occasionally displayed intracellular signals with varying patterns, including granular staining within the cell soma that extended into the axonal compartment and a limited perinuclear staining pattern (Fig. 2a). Notably, the frequency of cells exhibiting perinuclear signals varied depending on the antibody used. Morphologically, MCT8-ir neurons varied in size, shape, and signal intensity throughout all examined cortical regions (see Methods). Among the cortical layers, layer V stood out with a row of neurons with triangular-shaped cell bodies, whereas layers II-VI contained occasional MCT8-expressing neurons with pyramidal and round cell bodies. The laminar distribution and the criteria used to identify these cells are described below (Fig. 5e).

We performed additional immunostainings using the axonal marker NF-H and the somatodendritic marker MAP2 to assess MCT8-ir in axonal and dendritic neuronal compartments and to examine the long, fibre-like MCT8-ir structures observed in the juxtacortical and subcortical white matter. The NF-H-immunoreactive structures occasionally colocalized with MCT8 signals, suggesting MCT8 expression at the cell membrane and axons of large projecting neurons (Fig. 2b, NF-H). Similarly, we observed occasional colocalization of MAP2 and MCT8 signals, suggesting MCT8 expression within or on dendrites (Fig. 2b, MAP2). The immunosignals often exhibited a punctate appearance and were primarily located on the outer surface of the examined dendrites and axons (Fig. 2b).

To further characterize the observed MCT8-expressing neurons, we combined MCT8 with markers of defined neuronal populations. Co-staining for GAD67, an established marker of GABAergic neurons, identified occasional MCT8-ir cells that were simultaneously NeuN- and GAD67-positive, indicating that MCT8 is present in a subset of GABAergic neurons (Fig. 2c). We further observed MCT8-ir in neurons that were immunoreactive for THy (Fig. 2d). THy-expressing cells in the human cerebral cortex represent a distinct population of local circuit interneurons, approximately half of which contain GABA [7]. Because THy expression alone does not confirm complete catecholamine synthesis, we refer to these neurons as THy-immunoreactive alone. A catecholaminergic or even dopaminergic assignment would require additional markers [7]. Finally, we examined the relationship between MCT8-expressing neurons and glutamatergic terminals. VGLUT1-immunoreactive puncta were observed in close perisomatic apposition to MCT8-ir neurons (Fig. 2e). Consecutive optical sections of a z-stack confirmed that individual puncta remained in direct contact with, but separate from, the MCT8-positive somatic membrane (Fig. 2e, z-stack). Because VGLUT1 is confined to presynaptic terminals and not to the somata they contact [33], these images document apposition of glutamatergic terminals to MCT8-expressing neurons rather than co-expression of the two proteins. These findings suggest that MCT8-expressing neurons receive glutamatergic synaptic input.

### *SLC16A2* mRNA expression patterns and correlation with MCT8 protein

To map *SLC16A2* mRNA distribution across cell types and assess mRNA-protein concordance, we performed RNAscope using a probe specific for *SLC16A2* mRNA alongside cell-type-specific mRNA probes (*RBFOX3 and P2RY12*). We first validated the *SLC16A2* probe in the thyroid gland as a positive-control tissue, given the well-established expression of MCT8 in thyroid follicular cells [27, 62]. *SLC16A2* expression was detected in thyroid follicular cells, outlining the characteristic follicles (Fig. S4a–b, white arrow). In the adjacent endothelium, fewer *SLC16A2* probe signals were detected (Fig. S4a, white asterisk). We then combined RNAscope with standard IF labelling in formalin-fixed, paraffin-embedded hippocampus and postcentral gyrus tissue from two donors.

**Fig. 3:**
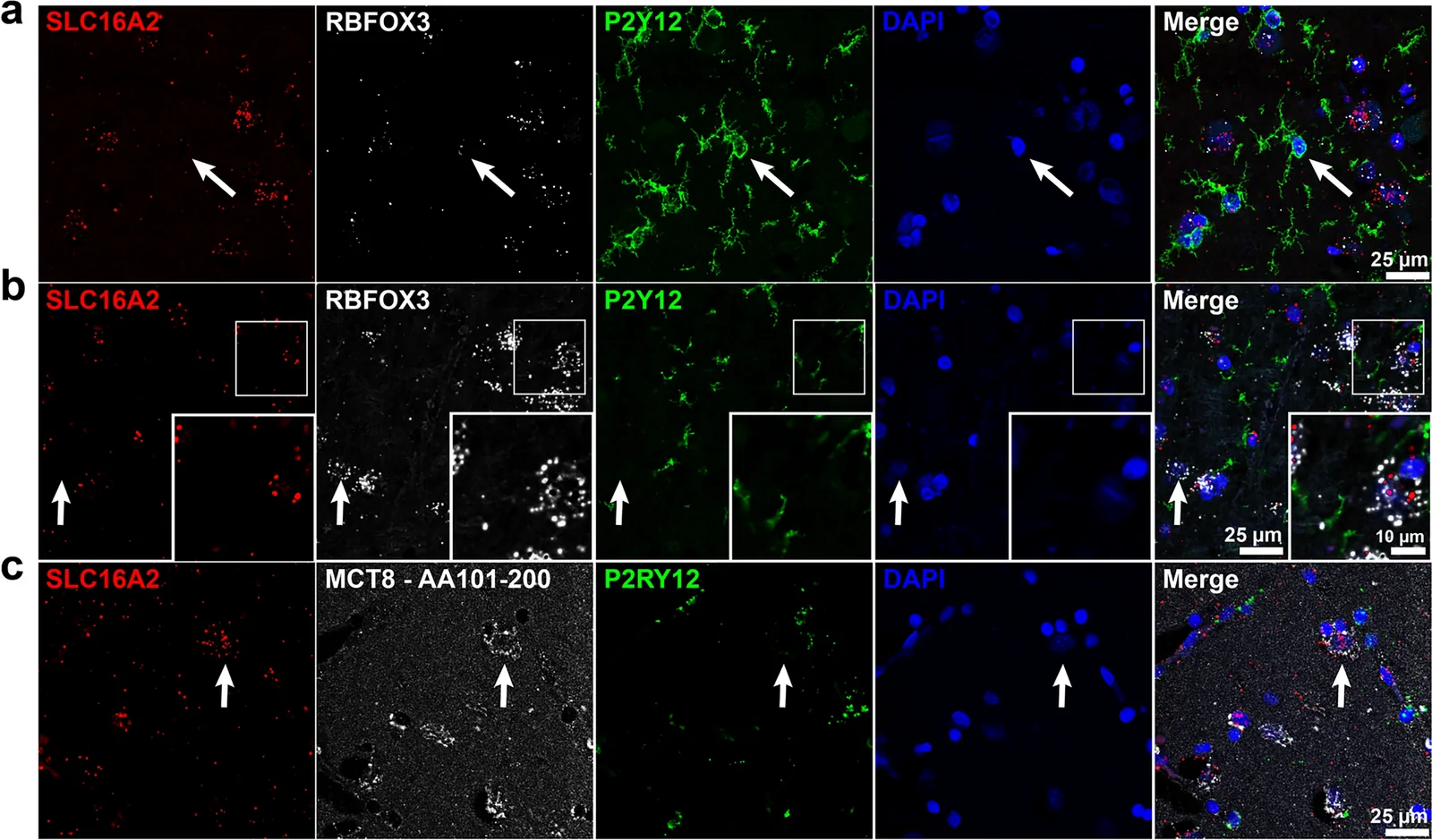
*SLC16A2* mRNA is present in neurons and colocalizes with MCT8 protein, but is not detected in microglia. Representative images of RNAscope combined with immunofluorescence staining in human hippocampus (n = 2 donors). Probes: *SLC16A2* (**a**–**c**, red), *RBFOX3* (**a**, **b**, white), *P2RY12* (**c**, green). Antibodies: P2Y12 (**a**, **b**, green), MCT8 (**c**, white, middle region antibody). Nuclei were labelled with DAPI (blue). Scale bars apply to all panels in the same row. Insets have their own scale bars. **a**, **b** Same triple-labelling experiment, shown at a microglial cell (**a**) and at neurons (**b**). **a** P2Y12-immunoreactive microglia showed no detectable *SLC16A2* probe signal in their somata (white arrow). **b** *SLC16A2* and *RBFOX3* signals colocalized in *RBFOX3*-positive cells (inset), confirming neuronal *SLC16A2* expression. Other *RBFOX3*-positive cells showed no detectable *SLC16A2* signal (white arrow). **c** MCT8 immunosignals and *SLC16A2* probe signals were detected in the same cells (white arrow), confirming the co-expression of transcript and protein. *P2RY12*-positive microglia in the same field showed no MCT8 immunosignal

Because MCT8 expression has previously been reported to be absent in human microglia [89], we first examined *SLC16A2* expression in these cells. Microglial-specific antibodies (Iba1, TMEM119, P2Y12) did not perform adequately in the fresh-frozen brain samples. We therefore combined RNAscope for *SLC16A2* and *P2RY12* with P2Y12 immunolabelling. Cells identified as microglia by P2Y12 antibody signals showed no *SLC16A2* probe signals in their somata (Fig. 3a, white arrow). RNAscope using probes against *SLC16A2* and *RBFOX3* demonstrated *SLC16A2* expression in *RBFOX3*-positive neurons (Fig. 3b, detail tile). Conversely, some *RBFOX3*-positive neurons did not display any *SLC16A2* mRNA signals (Fig. 3b, white arrow).

To assess the relationship between *SLC16A2* mRNA and MCT8 protein expression, we combined RNAscope for *SLC16A2* and *P2RY12* with IF for MCT8 in the same tissue sections. MCT8 immunosignals and *SLC16A2* probe signals were detected in the same cells (Fig. 3c), consistent with our antibody validation findings (Fig. S1). Overall, mRNA and protein signals showed good concordance, although not all *SLC16A2*-positive cells exhibited detectable MCT8 antibody signals.

### STED microscopy reveals MCT8 signals adjacent to the nuclear lamina

**Fig. 4:**
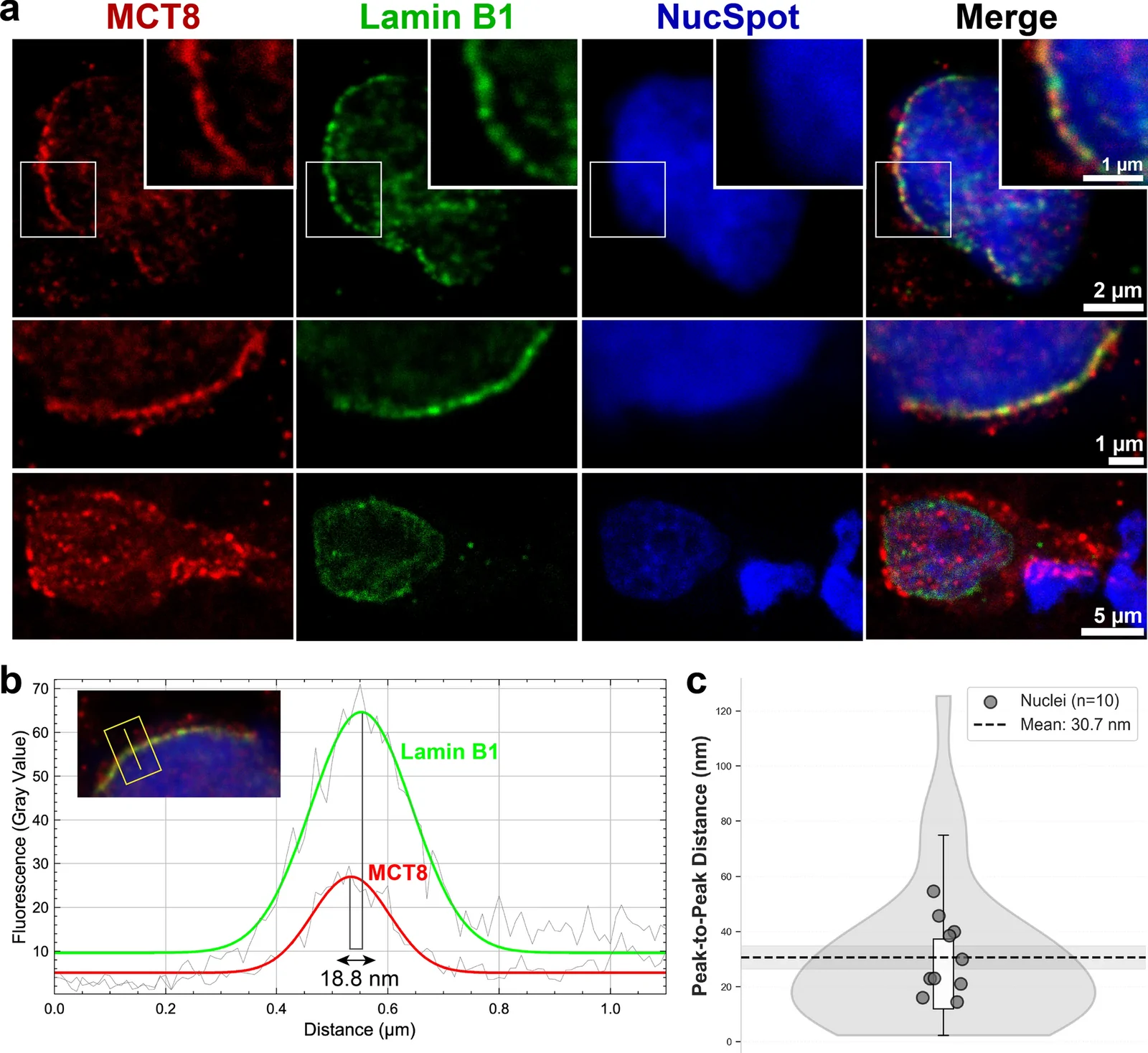
STED microscopy revealed the presence of MCT8 adjacent to the nuclear lamina. **a** Neurons of the temporal cortex were immunostained for MCT8 (red) and Lamin B1 (green), and counterstained with NucSpot (blue). Images were acquired using STED super-resolution microscopy. The two upper rows show staining with the middle-region MCT8 antibody and the lower row the polyclonal N-terminal antibody (see Methods). The IF signals suggest localization of MCT8 at or near the nuclear lamina. **b** Representative fluorescence intensity line profiles across the nuclear lamina. Peak positions of each signal were determined by Gaussian curve fitting to calculate the peak-to-peak distance between MCT8 and Lamin B1. **c** Distribution of peak-to-peak distances between MCT8 (middle-region antibody) and Lamin B1 fluorescence maxima across 10 nuclei from two samples. The violin plot shows the distribution of all individual measurements (n = 38); dots represent nucleus-level means. The embedded box plot indicates the median and interquartile range, with whiskers representing the data range. The dashed line indicates the overall mean (30.7 nm)

To resolve the distinct MCT8 immunolabelling patterns beyond the resolution limits of conventional confocal microscopy, we performed super-resolution stimulated emission depletion microscopy (STED) to characterize the subcellular location of MCT8. Given the observed perinuclear MCT8 signals, we employed an antibody against Lamin B1, a key structural component of the nuclear lamina, to determine the spatial relationship between MCT8 and the nuclear envelope. STED microscopy revealed a close spatial association between MCT8 and Lamin B1 signals, suggesting that MCT8 is not only present at the cellular membrane, but also near or at the nuclear lamina (Fig. 4a). To support our qualitative observations, we quantitatively analysed fluorescence intensity peaks across the intra- and extranuclear compartments for each channel (Fig. 4b) and calculated the distance between these peaks. By applying this analysis to a total of ten nuclei from two samples, we determined a mean distance of 30.7 nm between Lamin B1 and perinuclear MCT8 signals (Fig. 4c).

### Regional analysis reveals differential MCT8 expression across the human brain

**Fig. 5:**
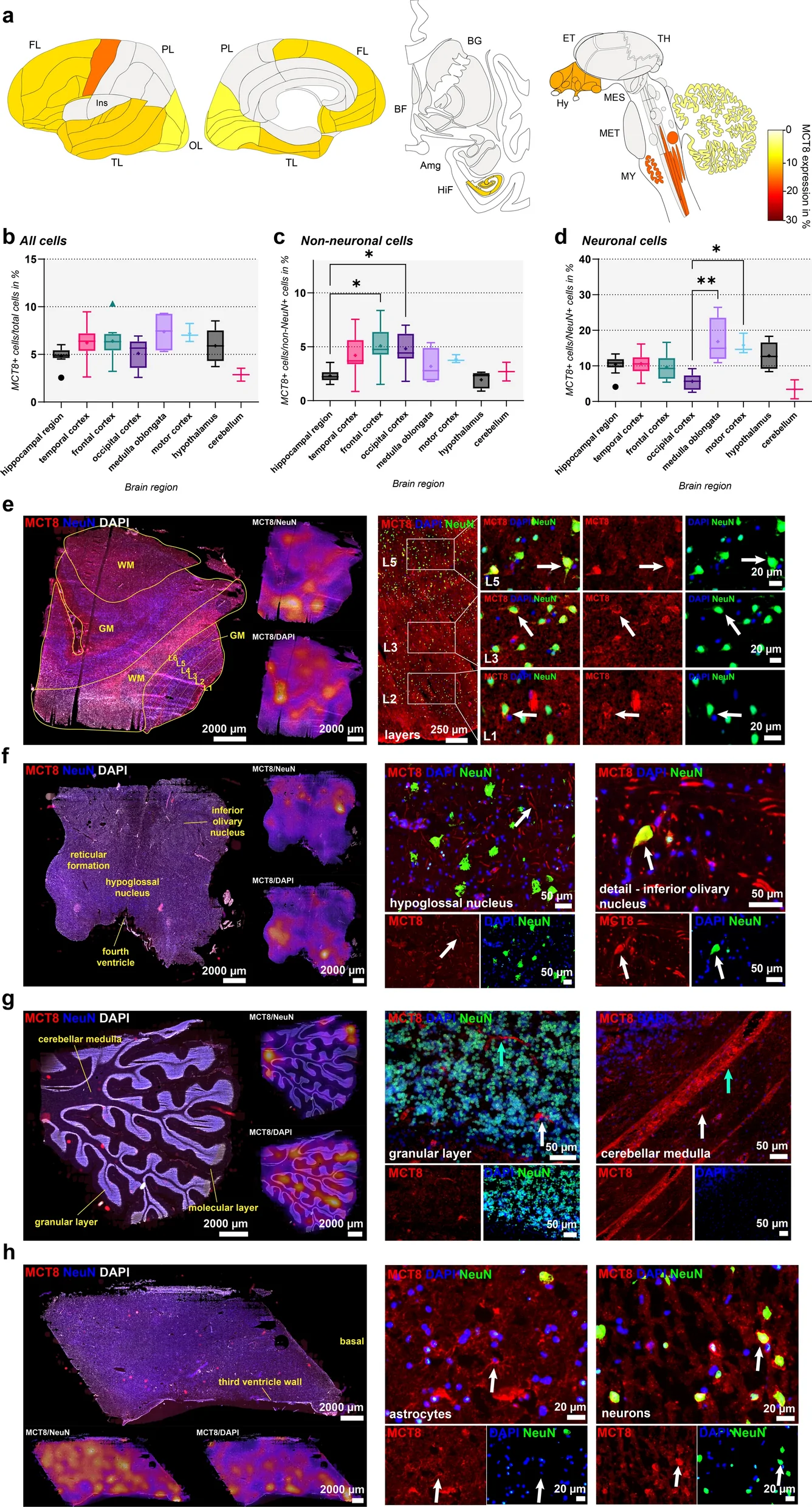
Regional MCT8 expression patterns differ between neuronal and non-neuronal cells. Human post-mortem brain samples from 14 individuals and eight brain regions were analysed for MCT8-expressing cells by whole-slide immunofluorescence for MCT8 (red), NeuN (blue) and DAPI (white). Non-neuronal cells were defined as DAPI-positive, NeuN-negative cells. Throughout, MCT8 expression denotes the proportion of MCT8-immunoreactive (MCT8-ir) cells among all detected cells of the respective cell class. **a** Overview of the analysed brain regions. The colour gradient encodes the mean proportion of MCT8-ir cells among NeuN-positive cells per region. Structures in grey were not analysed. **b** Boxplots depicting MCT8 expression across all cells, **c** non-neuronal (NeuN-negative) cells, and **d** neuronal (NeuN-positive) cells for each brain sample. Values were averaged per donor (hippocampal region n = 11, temporal cortex n = 10, occipital cortex n = 9, frontal cortex n = 8, hypothalamus n = 5, medulla oblongata n = 4, motor cortex n = 3, cerebellum n = 2). Boxes indicate the median and interquartile range, whiskers indicate the Tukey range (1.5× interquartile range), "+" denotes the arithmetic mean. Regional differences were assessed using Kruskal–Wallis tests followed by Dunn's multiple comparisons test. Only statistically significant comparisons are indicated. *p<0.05, **p<0.01. **e**–**h** Left: representative whole-slide overviews with anatomical annotations and density maps, in which MCT8/NeuN depicts the local density of MCT8-ir neurons and MCT8/DAPI the local density of MCT8-ir non-neuronal cells. Warmer colours indicate higher density. Right: higher-magnification details. **e** Temporal cortex. Left: Hotspots of MCT8-ir neurons occurred in layer V and hotspots of MCT8-ir non-neuronal cells occurred in the juxtacortical white matter. Right: cortical layer overview with detected MCT8-ir cells and higher-magnification details from layers V, III and II. MCT8-ir neurons spanned all cortical layers and exhibited heterogeneous morphologies. **f** Medulla oblongata. Right: Higher-magnification details of the hypoglossal nucleus and of the inferior olivary nucleus. MCT8-ir was mostly present in large axonal profiles and blood vessels (white arrow, left panel); individual neurons of the medullary nuclei (white arrow, right panel) were also MCT8-ir. **g** Cerebellum. Right: Granular layer and the cerebellar medulla detail views. MCT8-ir was restricted primarily to blood vessels (cyan arrows) and ramified astrocyte-like cells (white arrows). NeuN-positive/MCT8-positive neurons were rare. **h** Hypothalamus. MCT8-ir NeuN-negative cells were enriched adjacent to the third ventricle wall, whereas MCT8-ir neurons were distributed diffusely throughout the parenchyma. Right: NeuN-negative MCT8-ir cells with a ramified, astrocyte-like morphology (arrows, left) and MCT8-ir neurons (arrows, right). Scale bars are indicated in each image. *Abbreviations*: *FL* Frontal lobe, *Ins* Insula, *PL* Parietal lobe, *TL* Temporal lobe, *OL* Occipital lobe, *BF* Basal forebrain, *BG* Basal ganglia, *Amg* Amygdala, *HiF* Hippocampal formation, *ET* Epithalamus, *Hy* Hypothalamus, *TH* Thalamus, *MES* Mesencephalon, *MET* Metencephalon, *MY* Myelencephalon, *WM* White matter, *GM* Grey matter, *L1-L6* Layer I to Layer VI

To quantify and spatially map MCT8 expression throughout the human brain, we analysed brain samples from 14 individuals across eight different brain regions for MCT8-expressing cells (Fig. 5a), totalling 13,664,502 analysed cells. Density maps were generated to identify regional hotspots of MCT8 expression.

MCT8-expressing cells were detected in every examined region. However, none of the pairwise comparisons between the eight regions reached statistical significance (Fig. 5b). Descriptively, values were lowest in the cerebellum and highest in the medulla oblongata and the motor cortex/precentral gyrus. Analysing the neuronal and non-neuronal compartments separately, however, revealed distinct regional patterns. Within the non-neuronal class, MCT8 expression was lowest in the hypothalamus and the hippocampal region and highest in the frontal and occipital cortex, with the hippocampal region differing significantly from both the frontal (p = 0.023) and the occipital cortex (p = 0.025) (Fig. 5c). Within the neuronal compartment, expression was highest in the motor cortex and the medulla oblongata and lowest in the cerebellum and the occipital cortex, with the latter differing significantly from both the medulla oblongata (p = 0.004) and the motor cortex (p = 0.010) (Fig. 5d). The occipital cortex was thus among the regions with the highest non-neuronal expression and among the lowest for neuronal MCT8 expression, suggesting that regional variation in MCT8 expression is compartment-specific rather than uniform across cell classes. Expression in the cerebellum was lowest in both the neuronal and the all-cell analysis, although this comparison did not reach statistical significance.

In the temporal, frontal, occipital and motor cortices, MCT8-expressing cells were distributed across all cortical layers. Blood vessels throughout the cortical layers showed abundant MCT8-ir. MCT8-ir neurons were morphologically heterogeneous. Large MCT8-ir neurons with a triangular soma and prominent, apically oriented dendrites, morphologically consistent with pyramidal projection neurons, were observed across multiple cortical layers but were most prominent in layer V. Smaller MCT8-ir neurons were observed across all layers (Fig. 5e, temporal cortex). These neurons were identified based on their anatomical location and somatodendritic morphology. As we did not employ a pyramidal-neuron-specific marker, this assignment remains purely morphological. Consistent with these observations, density maps identified multiple hotspots of MCT8-ir neurons within the cortical grey matter, with layer V contributing most prominently to this enrichment. Non-neuronal hotspots were primarily associated with the juxtacortical white matter, large blood vessels and larger choroid plexus villi (Fig. 5e).

The medulla oblongata presented a staining pattern that differed from the other examined brain regions due to its unique anatomy, which contains both functional nuclei and a large number of fibre tracts. We observed MCT8-ir mainly in large axons and blood vessels, but also in neurons of the various nuclei of the medulla oblongata located between the individual axon strands, as seen in the inferior olivary nucleus (Fig. 5f). While the absolute number of neurons was considerably smaller here, the ratio of MCT8-positive to all neurons was among the highest of all examined regions (Fig. 5d).

In the cerebellum, we primarily found MCT8 signals in blood vessels of the molecular and granular layer and the cerebellar white matter (Fig. 5g). Occasional ramified cells with astrocyte-like morphology were observed in the granular layer and white matter, whereas only very few neurons were MCT8-positive (Fig. 5g, left and right detail panels).

The hypothalamus exhibited the most complex and heterogeneous MCT8 expression pattern among all investigated brain regions (Fig. 5h). While non-neuronal MCT8-expressing cells were concentrated at the surface facing the third ventricle, we also observed some MCT8-ir processes extending deep into the tissue (see also Fig. 1e). Within the parenchyma, MCT8-expressing neurons and astrocytes were distributed relatively evenly.

### Detailed characterization of MCT8 expression in the hippocampal formation

**Fig. 6:**
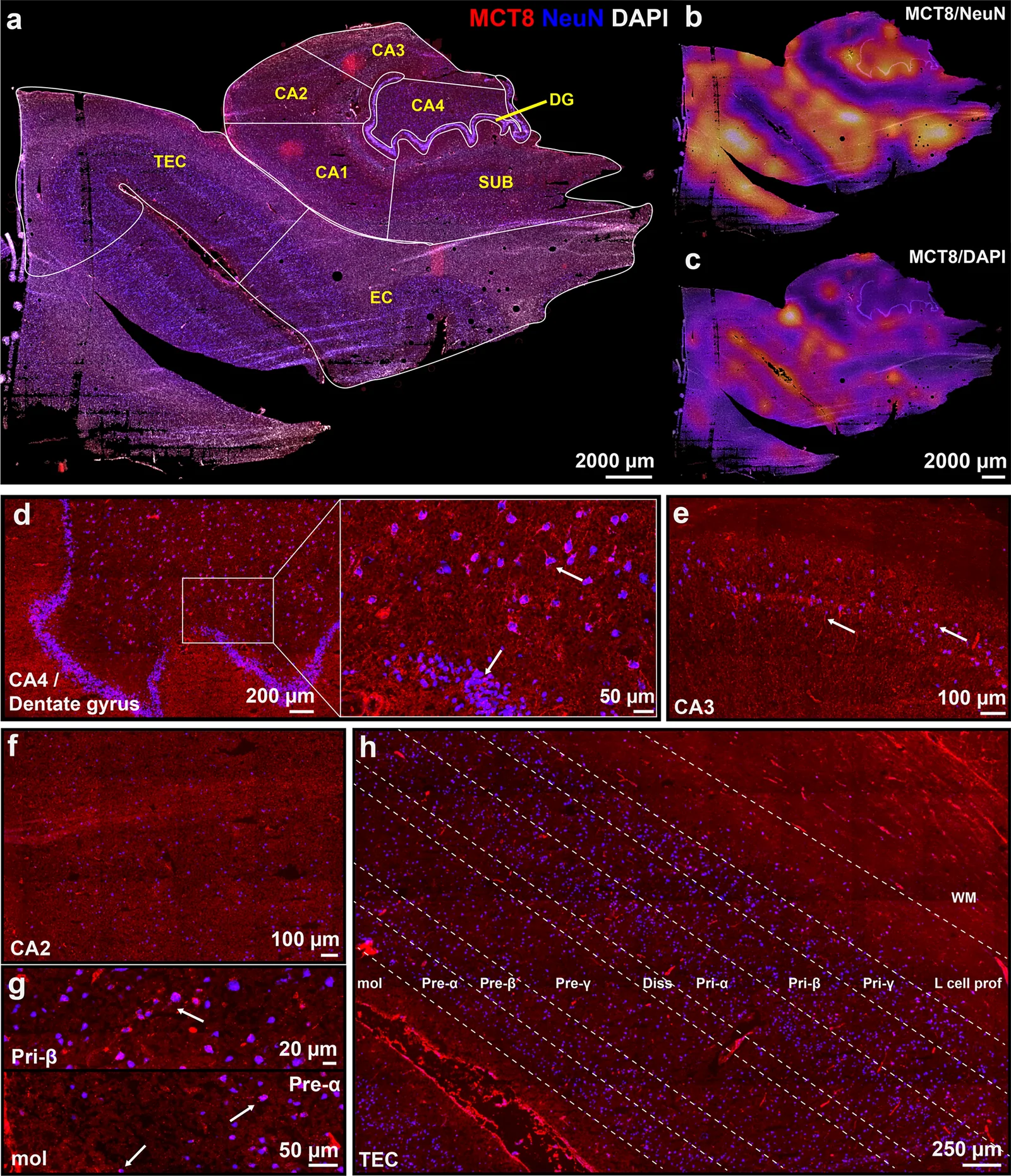
Heterogeneous MCT8 expression throughout the hippocampal formation. Automated high-resolution image analysis revealed a specific MCT8 expression pattern within the hippocampal formation and adjacent cortex. The analysis was performed on hippocampal tissue from 13 of the 14 donors. Representative images from one donor are shown. Scale bars are shown in each image. **a** Overview of the hippocampal formation and adjacent entorhinal and transentorhinal cortex, with subfields and entorhinal sublayers annotated. **b** Density map of classified MCT8-expressing cells relative to NeuN. **c** Density map of classified non-neuronal MCT8-expressing cells relative to DAPI. In (**b**) and (**c**), colour indicates relative local cell density, from low (dark blue/purple) to high (yellow to white). **d** In the DG and CA4, MCT8 immunosignals were prominent in both the granular cell layer and in triangular-shaped mossy cell-like neurons of the polymorphic layer (inset, white arrows). **e** In CA3, MCT8-positive neurons were smaller with long immunoreactive processes (white arrows). **f** MCT8 expression was comparatively lower in CA2 than in CA3. **g** Layer-specific differences in neuronal density in the pri-β (upper panel), molecular and pre-α (lower panel) layers, with a consistent proportion of MCT8-ir neurons (white arrows). **h** MCT8 expression was relatively uniform across the cortical layers of the TEC. MCT8 (red), NeuN (blue), DAPI (white). *Abbreviations*: *DG* Dentate gyrus, *CA* Cornu ammonis, *SUB* Subiculum, *EC* Entorhinal cortex, *TEC* Transentorhinal cortex, *mol* Molecular layer, *Pre-α* Layer Pre-α, *Pre-β* Layer Pre-β, *Pre-γ* Layer Pre-γ, *Diss* Lamina dissecans, *Pri-α* Layer Pri-α, *Pri-β* Layer Pri-β, *Pri-γ* Layer Pri-γ, *L cell prof* Lamina cellularis profunda, *WM* White matter

Whole-slide image visualization highlighted MCT8-ir in specific regions of the hippocampal formation (Fig. 6a–c). Density maps computed relative to NeuN-positive nuclei (Fig. 6b) and relative to all DAPI-positive nuclei (Fig. 6c) identified hotspots of MCT8 expression among neurons and across all cell types. In the dentate gyrus (DG), we observed robust MCT8 immunosignals within both the granular cell layer and the polymorphic layer (also known as the hilus of the DG or cornu ammonis 4 (CA4)) (Fig. 6d). Particularly striking were the triangular-shaped neurons in the polymorphic layer, which exhibited strong MCT8 signals across the entire plasma membrane of both the cell soma and projecting processes (Fig. 6d, inset). Given their anatomical location, large somata, and extensive cellular processes, these cells were morphologically consistent with hippocampal mossy cells. However, as we did not apply co-staining with a specific marker (e.g., GluR2/3), we cautiously refer to them as mossy cell-like neurons. We also observed elongated processes that lacked direct contact with neighbouring cells, displaying a granular or dotted staining pattern. These processes co-labelled with neurofilament markers, as depicted in Fig. 2b.

Tracing the mossy fibres from the DG hilus toward CA3, MCT8 immunosignals initially diminished, but increased again within in the middle and distal portions of CA3 (Fig. 6e). Neuronal MCT8 expression was lower in CA2 than in CA3 (Fig. 6f). Across these regions, MCT8-expressing neurons were heterogenous in both soma size and MCT8 immunosignal intensity. This pattern persisted through the subiculum and extended into the entorhinal cortex, where MCT8-expressing neurons were smaller in size. In the entorhinal and transentorhinal cortex, the proportion of MCT8-expressing neurons remained relatively stable across the cortical layers. Cell-poor layers such as the molecular layer contained only few MCT8-ir cells (Fig. 6g, mol), consistent with their low neuronal density, whereas the external (pre-α, pre-β, pre-γ) and internal (pri-α, pri-β, pri-γ) principal layers contained higher neuronal numbers and correspondingly more MCT8-expressing neurons (Fig. 6g, pri-β and pre-α). Consequently, the overall ratio of MCT8-expressing to non-expressing neurons appeared consistent throughout the cortical layers (Fig. 6 a, c, and h).

### snRNA-seq analysis of *SLC16A2* across brain regions and cell types

**Fig. 7:**
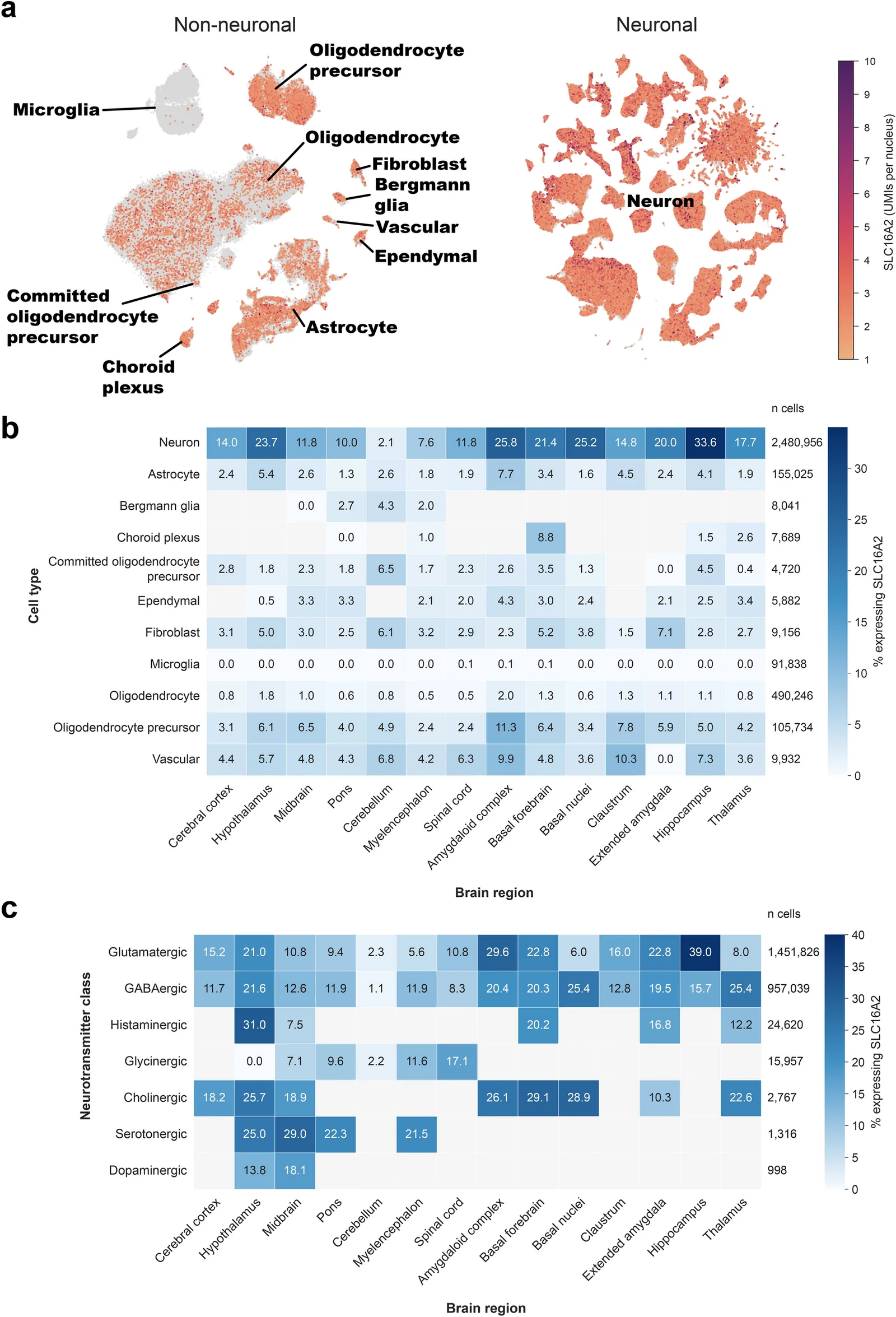
Single-nucleus RNA sequencing analysis of *SLC16A2* expression across brain regions, cell types, and neurotransmitter classes. Publicly available single-nucleus RNA sequencing data from the adult human brain, accessed through the Allen Brain Cell Atlas [75], were analysed for *SLC16A2* expression. **a** UMAP embeddings provided with the atlas for non-neuronal (left) and neuronal (right) nuclei, coloured by raw *SLC16A2* count. Grey denotes negative nuclei. Non-neuronal superclusters are labelled individually, the neuronal superclusters are summarized as "Neuron". Microglia formed the only supercluster displaying essentially no *SLC16A2*-positive nuclei. **b** Percentage of *SLC16A2*-positive nuclei for each combination of cell type and brain region. Rows correspond to the ten non-neuronal superclusters of the atlas and to all neurons combined. **c** Percentage of *SLC16A2*-positive nuclei for neurons grouped by neurotransmitter class. In (**b**) and (**c**), colour encodes the percentage of positive nuclei, values are printed in each field, and blank fields denote cell type–region combinations containing fewer than 20 nuclei, which were excluded from the analysis. The numbers at the right of each row indicate the total number of nuclei in each group across the whole dataset. *Abbreviations*: *OPC* Oligodendrocyte precursor cell, *UMAP* Uniform manifold approximation and projection, *UMI* Unique molecular identifier

To characterize the cell-type- and region-specific expression of *SLC16A2* across the adult human brain, we analysed a published snRNA-seq dataset [75] (Fig. 7). UMAP feature plots demonstrated broad expression of *SLC16A2* across both non-neuronal and neuronal superclusters, with microglia forming the only cluster essentially devoid of signal (Fig. 7a).

The proportion of *SLC16A2*-positive nuclei differed markedly between cell types and regions (Fig. 7b). Neurons showed the highest proportion in 13 of 14 regions, from 7.6% (myelencephalon) to 33.6% (hippocampus), with high values also in the amygdaloid complex (25.8%), basal nuclei (25.2%) and hypothalamus (23.7%). The cerebellum was the exception, with only 2.1% of neurons expressing *SLC16A2*, ranking below vascular cells (6.8%) and committed oligodendrocyte precursors (6.5%) in the same region. This reproduced our histological findings of rare MCT8-ir neurons but abundant MCT8-ir vessels in the cerebellum (Fig. 5g). Among non-neuronal cells, oligodendrocyte precursor cells (OPCs, 2.4–11.3%) and vascular cells (0.0–10.3%) showed the highest proportions of *SLC16A2*-positive nuclei, followed by astrocytes (up to 7.7%) and fibroblasts (up to 7.1%). Within the oligodendroglial lineage, mature oligodendrocytes were lower than OPCs (0.5–2.0%) in every region. Microglia were essentially negative throughout (≤ 0.1%), corroborating the absence of *SLC16A2* in *P2RY12*-positive microglia by RNAscope (Fig. 3a). Overall, the snRNA-seq data closely matched the regional and cellular distribution observed by immunohistochemistry.

Grouping neurons by neurotransmitter class showed that *SLC16A2* expression is diverse across the subgroups (Fig. 7c), similarly to our immunohistochemical results (Fig. 2c–e). Glutamatergic and GABAergic neurons showed comparable proportions across most regions, peaking in the hippocampus (39.0%) and thalamus/basal nuclei (25.4%), respectively. Modulatory neuronal classes showed *SLC16A2* expression within their anatomical distributions, including histaminergic neurons in the hypothalamus, serotonergic and dopaminergic neurons in the midbrain, cholinergic neurons in the basal forebrain, and glycinergic neurons in the spinal cord (Fig. 7c).

### MCT8 expression in subjects affected by Alzheimer’s disease

**Fig. 8:**
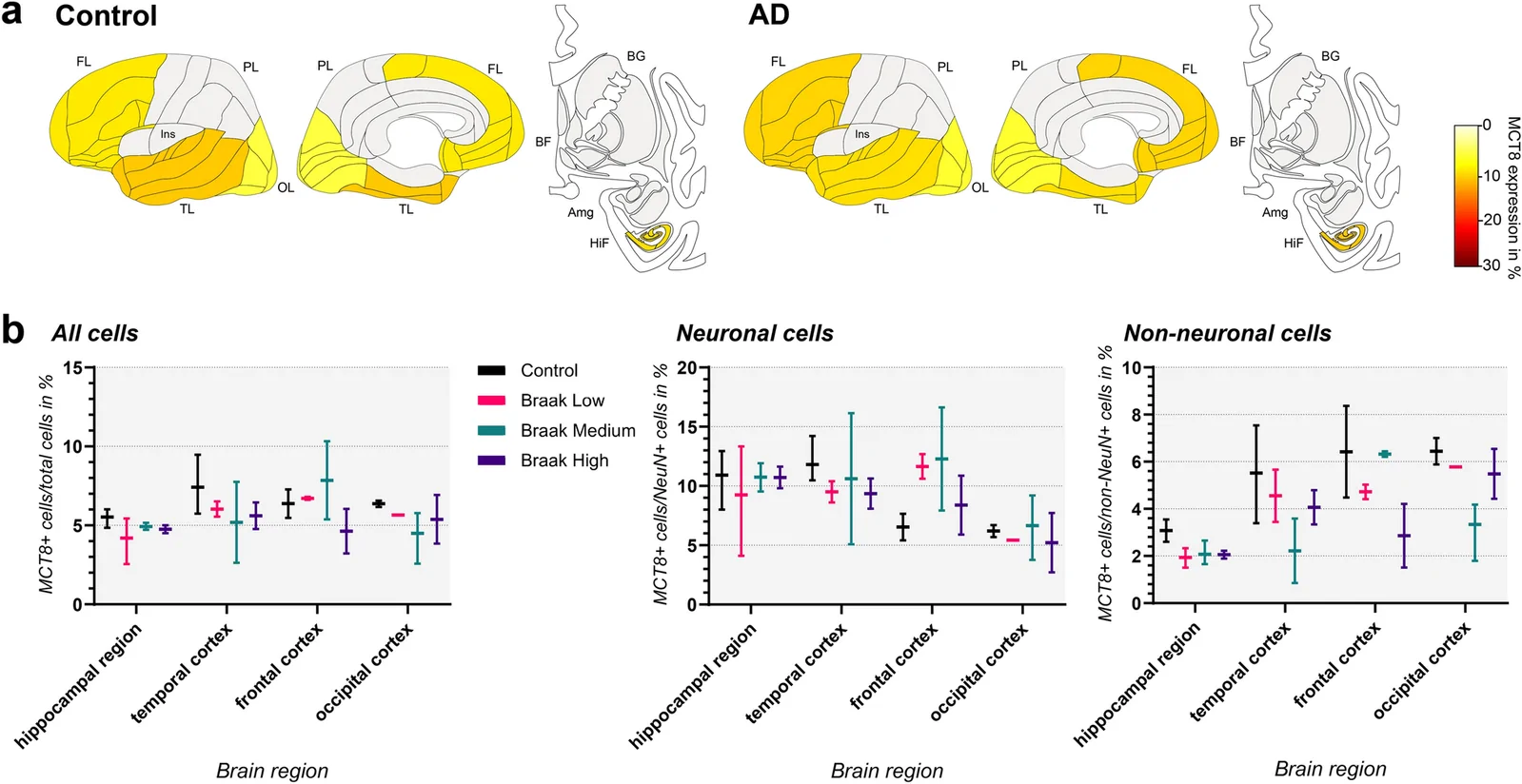
MCT8 expression remains stable across Braak stages. **a** Proportion of MCT8-positive cells among all NeuN-positive cells in the hippocampal formation and in the temporal, frontal and occipital cortex, compared between donors staged Braak 0–II ("Control" group, left) and Braak III–VI ("AD" group, right). Mann–Whitney U tests revealed no significant differences between the two groups in any region (all p ≥ 0.69). Analyses of all detected cells and NeuN-negative cells likewise revealed no significant regional differences. Group sizes varied by region (n = 3–6 donors per group), as not all regions were available from every donor. **b** Analysis of the same data resolved by the four Braak subgroups (Control, stages 0-I; Braak Low, stage II; Braak Medium, stages III-IV; Braak High, stages V-VI) and shown separately for all MCT8-positive cells, for MCT8-positive neuronal cells and for MCT8-positive non-neuronal cells. Two-way ANOVA comparing all subgroups revealed no significant differences. Interindividual variation in MCT8 expression was observed across all brain regions. Data are shown as group means (horizontal line) with whiskers indicating the range (n = 1–3 donors per subgroup). Braak subgroup assignment of individual donors is given in Table S1

To investigate whether neurofibrillary pathology influences MCT8 expression, we stratified our cohort according to Braak stage (see Methods). The analyses were restricted to the hippocampal formation and the temporal, frontal and occipital cortices, the regions represented across all Braak groups. Previously quantified MCT8 expression values were analysed as the proportion of MCT8-positive cells among NeuN-positive cells and, in parallel, relative to all detected cells and to NeuN-negative cells. Statistical comparisons between the two consolidated Control and AD groups (Fig. 8a) and across all four Braak subgroups (Fig. 8b) revealed no significant differences in MCT8 expression, regardless of cell class. Instead, we observed modest interindividual variability in MCT8 expression that appeared independent of neurofibrillary degeneration severity.

To complement our quantitative analysis, we visually inspected the whole-slide scans from all donors for MCT8 expression patterns differing between the Braak groups. No consistent differences were apparent between donors at low and high Braak stages. Finally, we employed double- and triple-immunostaining using MCT8, NeuN, and AT8 antibodies to examine the spatial relationship between MCT8-expressing cells and AT8-immunoreactive NFTs. These analyses revealed no clear colocalization or spatial association between MCT8 expression and neurofibrillary pathology.

## Discussion

Multiple studies have established the pivotal role of MCT8 in enabling early human brain development [3, 52]. However, while some evidence indicates its continued presence in adult motor components [88, 89], comprehensive data on its exact expression sites and mechanisms in the adult and aged human brain remain scarce. This raised the question motivating our study: does MCT8 expression persist across distinct regions in the adult and aged brain? Our data indicate that MCT8 forms a continuous anatomical route for TH transport from the brain barriers to individual parenchymal cells and, within these cells, from the plasma membrane potentially towards the nucleus. We also provide an anatomical framework that may help in understanding the motor, cognitive, and autonomic features of MCT8 deficiency.

### MCT8 expression at the brain barriers

MCT8 is expressed at brain barriers, which are a major site of restriction for TH entry in MCT8 deficiency experimental models [16, 85]. Whether they represent the principal limitation remains open: barrier-restricted deletion in mice does not fully reproduce the global knockout phenotype [2], human MCT8-deficient cerebral organoids show impaired T3 uptake and altered induction of TH-responsive genes [69], and OPCs require MCT8 at their own membrane [86]. Conversely, deletion restricted to murine GABAergic or glutamatergic neurons leaves hippocampus-dependent functions intact [1]. TH transporter expression at the brain barriers differs between rodents and humans [42, 67], limiting extrapolation from rodent models in either direction. In the human brain, our findings confirm that MCT8 is consistently expressed across all major brain barrier compartments, supporting their central role in cerebral TH transport.

### Regional MCT8 expression in the human brain

Recent findings by T. Wang and Y. Wang showed MCT8 expression in neurons of the motor cortex, the motor thalamus, and basal ganglia – brain regions which mediate voluntary motor function and are key regions affected by MCT8 deficiency [88, 89]. Y. Wang et al. suggested a key role of MCT8 in maintaining TH levels in these cells, underlining the importance of proper MCT8 function in the adult human brain and potentially explaining the loss of voluntary motor function in MCT8 deficient patients [89]. In the motor cortex, we likewise observed large, layer V neurons with triangular somata and prominent apical dendrites among the most intensely MCT8-ir cells. Their laminar position and morphology are consistent with pyramidal projection neurons, although we did not apply a pyramidal-neuron-specific marker. This pronounced expression suggests particularly high TH transport demands in these neurons. Consistent with the involvement of descending motor pathways, we also found one of the highest proportions of MCT8-expressing neurons in the medulla oblongata, a region critical for both motor output and autonomic regulation [45].

The clinical phenotype of MCT8 deficiency – including spasticity and childhood-onset parkinsonism, which manifest in characteristic features such as hypokinesia, dystonia and axial hypotonia – may reflect impaired TH signalling in the cortical and basal ganglia motor circuits where MCT8 expression is observed [88, 90]. The additional presence of bulbar symptoms and dysautonomia such as elevated resting heart rate and poor weight gain is consistent with the MCT8 expression we identified in the medulla oblongata [41, 73, 90]. In contrast, respiratory function and conjugated eye gaze are less severely affected, suggesting selective vulnerability of specific brainstem neurons to the loss of MCT8-mediated TH transport [28, 41]. The widespread MCT8 expression across motor cortex, basal ganglia, and brainstem regions is thus consistent with the multisystem neurological phenotype of MCT8 deficiency, underscoring the critical role of adequate TH transport for proper motor control and autonomic regulation throughout the central nervous system. These correspondences are anatomical correlates rather than explanations as our cohort is aged, whereas the phenotype of MCT8 deficiency originates in development.

### MCT8 expression in the hippocampus

A notable finding was the pronounced MCT8 expression in hippocampal granule and mossy cell-like neurons. While the exact function of these cells remains incompletely understood, they appear to be involved in neuroplasticity, spatial and associative learning, and pattern separation – processes vital for episodic memory formation [20, 23, 72]. If MCT8 maintains optimal levels of TH in these cells, its expression could indirectly enhance synaptic plasticity and support cognitive functions such as learning and memory [56].

Granule cells of the DG undergo adult neurogenesis, primarily in the subgranular zone [60]. The pronounced MCT8 expression we observed in these cells suggests a potential link to TH-dependent neurogenesis, as TH promote the proliferation and differentiation of neural stem cells [66]. While the extent and persistence of neurogenesis in the adult and aged human brain remains a subject of intense debate [76], impaired neurogenesis has been proposed as a candidate mechanism underlying cognitive decline, psychiatric disorders [30, 63] and AD [59]. Therefore, altered TH transport in the DG could theoretically contribute to cognitive impairment through this pathway. However, independent of neurogenesis, in mossy cells of the CA4 region, MCT8 may facilitate increased TH availability, supporting granule cell modulation and hippocampal output during memory processing. Additionally, TH-mediated regulation of glutamate receptor expression may further influence synaptic transmission and neuroplasticity [44].

Enhanced MCT8 expression was also observed in CA3, the subiculum, and the entorhinal and transentorhinal cortices – regions critically involved in episodic memory, spatial navigation, and cortico-hippocampal information integration [14, 84]. CA3 contributes to pattern completion via its recurrent circuitry, while the subiculum acts as a major hippocampal output relay [61, 64]. The entorhinal and transentorhinal cortices facilitate bidirectional communication between the hippocampus and neocortex, particularly during memory consolidation [15, 77]. The enriched presence of MCT8 in these areas suggests that TH transport via MCT8 may play a key role in supporting the metabolic and plasticity-related demands of these circuits, thereby contributing to the regulation of higher cognitive functions. Given the essential role of TH in neurodevelopment and synaptic function, impaired MCT8-mediated transport in these regions may contribute to memory deficits and disrupted hippocampal connectivity, as observed in patients with MCT8 deficiency [56, 73].

### MCT8 expression in AD affected patients

Despite the possible role of MCT8 in preserving and supporting cognition and memory function, our observation of consistent MCT8 expression levels in both AD and non-affected individuals suggests that MCT8 availability is not significantly altered by AD pathology. However, previous studies report altered TH metabolism in AD, potentially involving impaired local conversion (via deiodinases) and glial dysfunction [47, 51]. Subclinical thyroid dysfunction, which increases in prevalence with age [92], may additionally contribute to the altered TH levels reported in AD [71]. Moreover, alterations in the brain’s metabolic milieu or immunological alterations in AD may further modulate TH responsiveness or signalling. Notably, a recent study in an AD rat model showed that T3 supplementation restores cognitive function, with enhanced MCT8 expression facilitating hormone entry into the brain, possibly aided by improved cerebral blood flow [74]. These findings suggest that TH signalling deficits or dysregulation in AD likely results from altered hormone levels rather than MCT8 expression or insufficiency. While our data indicate that MCT8 expression is preserved across Braak stages and is therefore unlikely to drive TH dysregulation in AD, this does not exclude the converse possibility: individuals with constitutionally reduced MCT8 function, such as MCT8 mutation carriers, could in principle have lowered cerebral TH availability that increases vulnerability to, or accelerates the course of, age-related cognitive decline. This remains a hypothesis, as no direct clinical evidence currently links MCT8 mutation status to AD risk.

### Intracellular MCT8 expression

The concordance we observed between MCT8-ir and *SLC16A2* probe signals in hippocampal and postcentral gyrus cells is in line with previous observations of mRNA-protein correlation [52] and supports interpreting the observed intracellular signals as genuine, rather than as an antibody artefact. Previously, MCT8 was thought to exclusively reside at the cellular plasma membrane [35, 36]. More recently, Salas-Lucia et al. showed MCT8 to be expressed not only at the cellular plasma membrane, but also in endosomes, non-degrading lysosomes, the Golgi apparatus and in the nuclear membrane using electron microscopy in murine tissue [70]. These observations are consistent with our findings in human tissue, although our results were acquired with an imaging technique of lower resolution. Nonetheless, the mean MCT8-Lamin B1 peak-to-peak distance of 30.7 nm measured by STED microscopy supports a localization of MCT8 at or near the nuclear lamina. Salas-Lucia et al. further suggested that MCT8 contributes not only to TH uptake, but also to intracellular TH distribution and transport to and into the nucleus [70]. These findings may contribute to explaining the etiopathogenesis of the AHDS and challenges in medication trials to date [43]. We speculate that MCT8 may similarly facilitate intracellular TH transport in humans, ensuring TH delivery to and into the nucleus.

### MCT8 expression in oligodendrocytes and microglia

Wang et al. recently reported MCT8-ir fibres surrounded by MBP signal [89]. As MBP primarily labels the compact myelin sheath rather than the main oligodendrocyte cell body [5, 9], this indicates an association of MCT8 with myelinated fibres rather than expression in oligodendrocyte cell bodies. Our own MCT8/CNPase co-staining faces a similar limitation: CNPase localizes to the somata and non-compact myelin of differentiating and mature oligodendrocytes, but not OPCs [79], and is itself a myelin constituent, so the sparse co-labelling we observed cannot reliably assign MCT8 to the oligodendrocyte soma rather than to the surrounding sheath. Whether mature oligodendrocytes express MCT8 protein therefore remains unresolved by either approach. At the transcript level, *SLC16A2* was enriched in OPCs relative to mature oligodendrocytes, in line with the cell-autonomous requirement for MCT8 in OPCs [86], while remaining detectable at low levels in the mature population. Direct spatial transcriptomic validation of *SLC16A2* in human oligodendrocytes via multiplex RNAscope represents a valuable direction for future study.

The clinical relevance of MCT8 or *SLC16A2* expression in the oligodendroglial lineage remains debatable. Although conventional MRI in individual case reports has suggested an apparent normalization of myelination with age [65, 78, 82], post-mortem and histological studies show persistent myelin deficits, including reduced myelin staining and a shift toward small-calibre axons [53, 81]. This indicates that the hypomyelination does not truly resolve. Whether these deficits contribute directly to the permanent neurological phenotype of MCT8 deficiency or represent one manifestation of a broader central hypothyroid state affecting multiple cell populations, remains unresolved [73, 87].

Microglia, by contrast, showed no detectable *SLC16A2* by either RNAscope or snRNA-seq analysis. This aligns with prior reports that human microglia express OATP1C1 rather than MCT8 [89]. Consequently, microglial TH uptake in the adult human brain seems to operate independently of MCT8, an important fact, given the role of TH signalling in microglial immune function [47].

### Limitations of this study

A primary limitation of this study is the relatively narrow age range of our post-mortem cohort (64 to 101 years). The expression of TH-handling proteins, plausibly including MCT8, is dynamic across development [8, 52], and murine data indicate an age-related decline in neuronal MCT8 expression [91]. Our immunohistochemical findings therefore mainly characterize MCT8 in the aged human brain and should not be extrapolated to younger adults, children, or the developmental period. Consequently, while our data provide a strong anatomical framework, inferences regarding the developmental origins of the MCT8 deficiency phenotype are necessarily indirect. Furthermore, due to the nature of post-mortem material, our work is restricted to descriptive spatial analysis of MCT8 distribution and cannot capture precise functional roles or expression dynamics. Our Braak-stratified comparison is similarly limited by small subgroup sizes, which permitted detection of only large effect sizes. Moreover, donors were staged for neurofibrillary pathology only, meaning that cases in the Braak III–IV range may represent primary age-related tauopathy rather than AD [21].

While our multiscale approach encompassed a broad range of functionally distinct brain regions, it did not include the subventricular zone (SVZ). Given the SVZ’s critical role as a lifelong neurogliogenic niche, investigating local MCT8 distribution and expression in this region represents an important possibility for future research. Relatedly, our Braak-stratified analysis was performed at region level and does not resolve effects restricted to individual hippocampal subfields, including the DG as a second neurogliogenic niche.

Finally, several cell-type-selective antibodies failed to yield interpretable signals in the used fresh-frozen tissue and were therefore omitted (Table S2). Additionally, using automated analysis of IF stainings can introduce variability through potential over- or under-detection of labelled cells. To mitigate this issue, we conducted repeated, partially supervised analyses; however, we cannot entirely exclude the possibility of inaccurate cell detection. To minimize any further technical or interindividual variability, all samples were processed using standardized protocols upon arrival in our institute.

### Synopsis of the physiological MCT8 expression model

Our data suggest that:

(i) MCT8 is expressed in a variety of regions in the adult and aged human brain.
(ii) MCT8 expression ensures adequate TH supply via a transport cascade from the BBB to individual neurons.
(iii) MCT8 contributes to transmembrane and intracellular transport of TH.
(iv) Physiological MCT8 expression in the human brain might be essential for maintaining normal cognitive performance, motor coordination, and autonomic regulation.

**Fig. 9:**
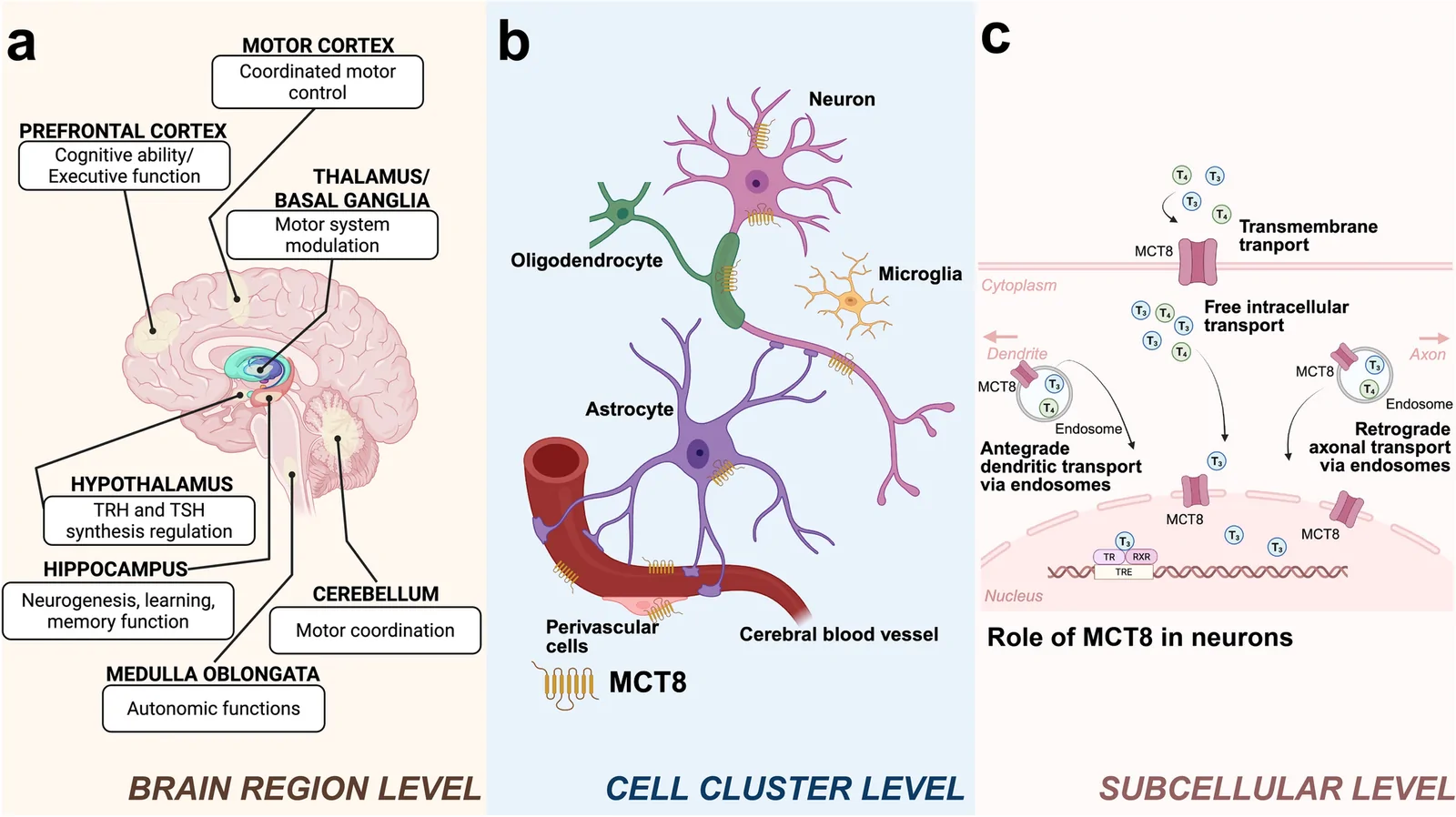
Perspectives on MCT8 expression in the human brain. **a** MCT8 is expressed across multiple brain regions, including the hippocampus, hypothalamus, and both cortical and subcortical areas. The regional distribution and clinical findings in MCT8-deficient patients indicate that sufficient MCT8 expression is essential for diverse neurological functions. Its widespread presence in the brain tissue suggests ongoing physiological relevance for these functions beyond neurodevelopment. **b** At the cellular level, MCT8 mediates TH transport between various cell types – such as endothelial cells, perivascular cells, astrocytes, and neurons – while being notably absent from microglia. **c** Subcellularly, MCT8 localizes to the plasma membrane and intracellular compartments, particularly near or at the nuclear lamina, suggesting roles in membrane-bound, intracellular, and possibly nuclear TH transport, potentially via endosomal pathways. *Created in BioRender. Rotter, J. (2026) (*https://BioRender.com/s08esrp*, *https://BioRender.com/qa325ul* and *https://BioRender.com/2o10zh3*)*

Overall, we present the most comprehensive analysis of MCT8 expression across the human brain to date, demonstrating its widespread distribution (Fig. 9). The observed expression pattern is consistent with previous findings and provides novel insights into brain regions where MCT8 expression has not been described before. The broad distribution of MCT8 in the human brain suggests a critical role not only in neurodevelopment, but also in maintaining TH homeostasis in the mature brain. By providing an anatomical framework, our findings may also help to explain some of the functional impairments observed in patients with MCT8 deficiency. Notably, we did not detect alterations in MCT8 expression under pathophysiological conditions. Our data therefore indicate that the previously reported altered TH levels in AD onset and progression are unlikely to result from changes in MCT8 expression.

## Supplementary Information

Supplementary Material 1.

## Data Availability

All code used for the analyses is available at the following data repository: https://github.com/jonrot1906/mct8-expression-human-brain. The image data for the automatic analysis of MCT8 expression is available from the corresponding author on reasonable request. The snRNA-seq data analysed for Fig. 7 are publicly available and were obtained from the Human Brain Cell Atlas [77] via the Allen Brain Cell Atlas (dataset WHB-10Xv3, manifest version 20260415; https://alleninstitute.github.io/abc_atlas_access/descriptions/WHB-10Xv3.html), accessed using the abc_atlas_access toolkit (https://github.com/AllenInstitute/abc_atlas_access).
