# Supplementary material for: Multiscale characterization and distribution of the thyroid hormone transporter MCT8 in the adult and aged human brain
Source: Acta Neuropathol Commun. 2026 Oct 1;14:200. doi: 10.1186/s40478-026-02435-3 (PMC13632270; doi:10.1186/s40478-026-02435-3)
Supplement: Supplementary file 1 [file 40478_2026_2435_MOESM1_ESM.docx]

**Multiscale characterization and distribution of the thyroid hormone transporter MCT8 in the adult and aged human brain**

Jonas Rotter^1^, Toni Kirmann^2^, Jana Brendler^1^, Larissa Anthofer^1,3^, Nina Maria Wilpert^4,5,6^, Stefan Hallermann^2^, Heike Biebermann^3^, Ingo Bechmann^1,7^

^1^Institute of Anatomy, Leipzig University, Leipzig, Germany.

^2^Carl-Ludwig-Institute of Physiology, Leipzig University, Leipzig, Germany.

^3^Charité Universitätsmedizin Berlin, Corporate Member of Freie Universität Berlin and Humboldt-Universität zu Berlin, Institute for Experimental Paediatric Endocrinology, Berlin, Germany.

^4^Department of Paediatric Neurology, Charité-Universitätsmedizin Berlin, Corporate Member of Freie Universität Berlin, Humboldt-Universität zu Berlin, and Berlin Institute of Health (BIH), Berlin, Germany.

^5^NeuroCure Cluster of Excellence, Charité-Universitätsmedizin Berlin, Corporate Member of Freie Universität Berlin, Humboldt-Universität zu Berlin, and Berlin Institute of Health (BIH), Berlin, Germany.

^6^Berlin Institute of Health at Charité - Universitätsmedizin Berlin, BIH Biomedical Innovation Academy, BIH Charité Junior Clinician Scientist Program, Berlin, Germany.

^7^LeiCeM – Leipzig Center of Metabolism, Leipzig University, Leipzig, Germany.

*Corresponding author: Jonas Rotter (J.R.)

E-mail: [jonas.rotter@medizin.uni-leipzig.de](mailto:jonas.rotter@medizin.uni-leipzig.de)

Mobile: +49 173 1925111

# **Supplementary Appendix**

# **Supplementary Tables**

**Table S1 Donor data.**

| **Case ID** | **Age/sex** | **PMI** | **NFT stage [1]** | **Assigned group** | **Status*** | **ICD/comorbidities** |
| --- | --- | --- | --- | --- | --- | --- |
| 1 | 94/m | 13 h | 2 | Braak Low | ND | I50.14, I35 |
| 2 | 95/m | 25 h | 4 | - | CD | I25.9, I10.0, F03 |
| 3 | 64/f | 27 h | 1 | Control | ND | A41.8, K65.0, K55.0, I70.8, I50.1 |
| 4 | 88/m | 28 h | 3 | Braak Medium | ND | I50.0, I25.1, I70.9 |
| 5 | 82/m | 23 h | 0 | Control | ND | D62, C67.2, R31, I25.19, E11.90 |
| 6 | 87/m | 15 h | 0 | Control | ND | I50.1, I25.9 |
| 7 | 97/f | 27 h | 5 | Braak High | CD | I50.9, I10.0, N18.5, F03 |
| 8 | 101/f | 19 h | 5 | Braak High | CD | I99, E46, F03, I10.0 |
| 9 | 95/f | 36 h | 3 | - | ND | I50.0, I35.0, E11.7, N18.4 |
| 10 | 84/m | 24 h | 4 | Braak Medium | CD | U07.1, F00.9 |
| 11 | 88/m | 17 h | 3 | Braak Medium | ND | I49.9, I21.9, I25.13, I71.8, I10.90 |
| 12 | 83/f | 27 h | 4 | - | CD | N17, N18.1, I10.9, U07.1, S72.10, F01.9 |
| 13 | 86/f | 24 h | 2 | Braak Low | ND | I50.19, A41.9, M86, I10.9, E11.8 |
| 14 | 88/m | 20 h | 2 | Braak Low | ND | I67.7, I63.5, I10.0, L89.3, I25.1, I70.2, N18.3 |

*CD: clinical dementia, ND: non-demented;

*Abbreviations and Explanations: PMI - post-mortem interval; - : not used for Braak stage comparison*

**Table S2 Primary Antibodies used in this study**

| **Antibodies** | **Structure** | **Host** | **Manufacturer** | **Number** | **Dilution** |
| --- | --- | --- | --- | --- | --- |
| Anti-MCT8 Polyclonal | MCT8 (TH transporter), N-terminal | Rabbit | Sigma-Aldrich / Atlas | HPA003353 | 1:500 |
| Anti-MCT8 Polyclonal | MCT8 (TH transporter), C-terminal | Rabbit | Novus | NBP2-57308 | 1:500 |
| Anti-MCT8 Polyclonal | MCT8 (TH transporter), middle region (AA101-200) | Rabbit | Antibodies online | ABIN1387834 | 1:100 |
| Anti-MCT8 Monoclonal [EPR26257-174] | MCT8 (TH transporter) | Rabbit | Abcam | ab302706 | 1:500 |
| Anti-Collagen Type IV Polyclonal | Collagen Type 4 (basement membrane) / Endothelial cell marker | Goat | Merck Millipore | AB769 | 1:1000 |
| Anti-GFAP | Glial fibrillary acid protein / Fibrous astrocyte marker | Mouse | BD Pharmingen | 556330 | 1:50 |
| Anti-Vimentin | Vimentin | Chicken | Merck Millipore | AB5733 | 1:300 |
| Anti-P2Y12 Polyclonal *(used on FFPE tissue only)* | Purinergic receptor P2Y G protein-coupled 12 (P2RY12) / Microglia marker | Rabbit | Sigma-Aldrich | HPA014518 | 1:500 |
| Anti-NeuN | Neuronal nucleus / Neuron marker | Guinea pig | Synaptic Systems | 266-004 | 1:200 |
| Anti-NeuN [A60] | Neuronal nucleus / Neuron marker | Mouse | Merck Millipore | MAB377 | 1:500 |
| Anti-MAP2 | Microtubule-associated protein 2 / Neuron marker | Mouse | Sigma | M4403 | 1:500 |
| Anti-Neurofilament H Polyclonal | Neurofilament H | Guinea Pig | Synaptic Systems | 171 104 | 1:500 |
| Anti-Lamin B1 | Lamin B1/Nuclear membrane | Mouse | Abcam | ab8982 | 1:500 |
| Anti-VGLUT1 Polyclonal | Vesicular Glutamate transporter 1 | Guinea Pig | Synaptic Systems | 135 304 | 1:500 |
| Anti-Tyrosine Hydroxylase Polyclonal | Tyrosine Hydroxylase | Sheep | Merck Millipore | AB1542 | 1:500 |
| Anti-GAD67 | Glutamic acid decarboxylase 67 | Mouse | Merck Millipore | mab5406 | 1:2500 |
| Anti-CNPase [11-5B] | CNPase | Mouse | Merck Millipore | MAB326 | 1:500 |
| Anti-Phospho-Tau [AT8] | Phospho-Tau (Ser202, Thr205) | Mouse | Thermo Fisher Scientific | MN1020 | 1:500 |

**Table S2 Primary Antibodies used in this study (continued)**

| **Tested antibodies (Unsuccessful in fresh frozen tissue)** | | | | | |
| --- | --- | --- | --- | --- | --- |
| Anti-CD140b / PDGFRB | Platelet-derived growth factor receptor beta | Rat | ebiosciences | 14-1402-82 | 1:100 |
| Anti-CD13 / APN | Aminopeptidase N | Rat | Acris | BM4009 | 1:100 |
| Anti-Iba1 | Ionized Calcium-binding Adapter Molecule 1 / Microglia and macrophage marker | Rabbit | Synaptic Systems | 234013 | 1:500 |
| Anti-Iba1 | Ionized Calcium-binding Adapter Molecule 1 / Microglia and macrophage marker | Guinea Pig | Synaptic Systems | 234004 | 1:500 |
| Anti-TMEM119 | TMEM119 / Microglia marker | Rabbit | Abcam | ab185333 | 1:1000 |
| Anti-Olig 2 (EP112) | Olig2 / Oligodendrocyte marker | Rabbit | Cell Marque | 387-R14 | 1:100 |
| Anti-Olig2 [EPR2673] | Olig2 / Oligodendrocyte marker | Rabbit | Abcam | ab109186 | 1:100 |
| Anti-Parvalbumin | Parvalbumin | Mouse | Abcam | ab277625 | 1:1000 |

**Table S3 Secondary Antibodies and TSA fluorophores used in this study**

| **Method** | **Antibodies** | **Host** | **Manufacturer** | **Number** | **Dilution** |
| --- | --- | --- | --- | --- | --- |
| IF | Alexa 488 Donkey anti Mouse | Donkey | Invitrogen | A21202 | 1:250 |
| IF | Alexa 488 Donkey anti Guinea Pig | Donkey | Jackson | 706-545-148 | 1:250 |
| IF | Alexa 568 Donkey anti Rabbit | Donkey | Invitrogen | A10042 | 1:250 |
| IF | Alexa 647 Donkey anti Mouse | Donkey | Invitrogen | A31571 | 1:250 |
| IF | Alexa 647 Donkey anti Guinea Pig | Donkey | Jackson | 706-605-148 | 1:250 |
| IF | Alexa 647 Donkey anti Goat | Donkey | Invitrogen | A21447 | 1:250 |
| IF | Alexa 647 Donkey anti Sheep | Donkey | Jackson | 713-606-008 | 1:250 |
| IF | Alexa 488 Goat anti Mouse | Goat | Invitrogen | A11029 | 1:250 |
| IF | Alexa 568 Goat anti Rabbit | Goat | Invitrogen | A11036 | 1:250 |
| IF | Alexa 647 Goat anti Chicken | Goat | Invitrogen | A21449 | 1:250 |
| RNAscope | Opal 570 |  | ACD | OP-001003 | 1:750 |
| RNAscope | Opal 690 |  | ACD | OP-001006 | 1:750 |
| STED | abberior STAR ORANGE Goat anti Rabbit | Goat | abberior | STORANGE-1002-500UG | 1:200 |
| STED | abberior STAR RED Goat anti Mouse | Goat | abberior | STRED-1002-500UG | 1:200 |

*Abbreviation: TSA – tyramide signal amplification*

**Table S4 RNAscope probes used in this study**

| **Probes** | **Gene** | **Target Region (bp):** | **Manufacturer** | **Number** |
| --- | --- | --- | --- | --- |
| RNAscope™ Probe- Hs-*SLC16A2* | *SLC16A2* | 1010 - 1984 | ACDbio | 562191 |
| RNAscope™ Probe- Hs-*RBFOX3*-C3 | *RBFOX3* | 720 - 2217 | ACDbio | 415591-C3 |
| RNAscope™ Probe- Hs-*P2RY12*-C2 | *P2RY12* | 121 - 1283 | ACDbio | 450391-C2 |

**Table S5 Nuclei labelling reagents used in this study**

| **Nuclei Labelling** | **Manufacturer** | **Number** | **Dilution** |
| --- | --- | --- | --- |
| DAPI | Sigma Aldrich | D9542 | 1:10000 |
| NucSpot LIVE 488 | Biotium | 40081-T | 1:1000 |

# **Supplementary Figures**

**Fig. S1 Validation of the MCT8 antibodies used in this study in wild-type and MCT8-knockout mouse brain.**

All four MCT8 antibodies used in this study (Table S2) were tested on brain tissue from MCT8-knockout (KO) mice and wild-type (WT) littermates. Columns in panels **(a-c)** show, from left to right, the C-terminal polyclonal (Novus NBP2-57308), the N-terminal polyclonal (Sigma-Aldrich HPA003353), the middle-region polyclonal (AA101-200; Antibodies-online ABIN1387834) and the N-terminal monoclonal antibody (Abcam ab302706). The upper row of panels **(a-c)** is WT tissue, the lower row KO. **(a)** Low-magnification overviews of coronal half-brain sections. White arrows mark the choroid plexus of the lateral ventricle. **(b)** Higher magnification of the choroid plexus. **(c)** Higher magnification of parenchymal blood vessels. **(d)** Omission control for the Alexa Fluor 568 donkey anti-rabbit secondary in WT brain tissue. In WT tissue, all four MCT8 antibodies labelled the choroid plexus and cerebral blood vessels (red, **a-c**). None gave a detectable signal in KO tissue. Omission controls were run alongside every primary antibody and gave no specific signal (d). Nuclei were counterstained with DAPI (blue). Staining performance in human brain tissue is compared in Fig. S2. Scale bars apply to all panels in the same row, for **(b)** and **(c)** for all images.

**Fig. S2 Comparison of the staining performance of the MCT8 antibodies used in this study in human brain tissue.**

All four MCT8 antibodies (Table S2) were applied to at least five brain regions (temporal, prefrontal, occipital cortex, hypothalamus and hippocampus) and at least five donors and compared qualitatively. Representative images are shown here. Each row shows the MCT8 signal (red), the DAPI counterstain (blue) and the merge. Boxed areas are shown enlarged as insets. (**a)** Polyclonal C-terminal antibody, prefrontal cortex. **(b)** Polyclonal N-terminal antibody, temporal cortex. **(c, d)** Polyclonal middle region antibody, prefrontal cortex. **(e)** Monoclonal N-terminal antibody, temporal cortex. Note the epitope-dependent differences in subcellular signal distribution. Scale bars apply to all panels in the same row. Insets have their own scale bars.

**Fig. S3 Workflow for automated whole-slide quantification of MCT8-expressing cells.**

A total of 78 slides were scanned using a high-resolution whole-slide scanner and analysed in QuPath. A custom image analysis script incorporated the deep learning tool StarDist for nuclei segmentation, estimated cell boundaries, and quantified intracellular MCT8 signal to classify cells as MCT8-positive or -negative. Raw data were exported in GeoJSON format and further processed using a Python-based pipeline to create brain-region based graphs and heatmaps. Full details are given in Methods, "Microscopy, semi-quantification and statistical analysis". *Created in BioRender. Rotter, J. (2026)* [*https://BioRender.com/wn3hy3z*](https://BioRender.com/wn3hy3z)

**Fig. S4 RNAscope in the human thyroid gland confirms *SLC16A2* mRNA in follicular epithelial cells (positive-control tissue).**

Thyroid gland tissue was used as a positive-control tissue to validate the *SLC16A2* probe, given the well-established expression of MCT8 in thyroid follicular cells. Representative images shown (n = 1 donor). **(a)** RNAscope using an *SLC16A2* probe (red) in human thyroid gland tissue shows punctate SLC16A2 mRNA signal within follicular epithelial cells lining the follicles (white arrow). In the inset, endothelial cells of an adjacent vessel show comparatively fewer SLC16A2 expression (white asterisk in the inset). **(b)** A second field of thyroid follicles, again with punctate *SLC16A2* signals in follicular cells (white arrow). Single channels are shown for DAPI (blue) and *SLC16A2* mRNA (red) alongside the merge. Representative images of formalin-fixed, paraffin-embedded human thyroid gland from one donor. Scale bars apply to all panels in the same row; insets have their own scale bars. **References**

1.	Braak H, Braak E (1991) Neuropathological stageing of Alzheimer-related changes. Acta Neuropathol 82:239–259. doi: 10.1007/BF00308809
